# A RecA Protein Surface Required for Activation of DNA Polymerase V

**DOI:** 10.1371/journal.pgen.1005066

**Published:** 2015-03-26

**Authors:** Angela J. Gruber, Aysen L. Erdem, Grzegorz Sabat, Kiyonobu Karata, Malgorzata M. Jaszczur, Dan D. Vo, Tayla M. Olsen, Roger Woodgate, Myron F. Goodman, Michael M. Cox

**Affiliations:** 1 Department of Biochemistry, University of Wisconsin-Madison, Madison, Wisconsin, United States of America; 2 Departments of Biological Sciences and Chemistry, University of Southern California, Los Angeles, California, United States of America; 3 Biotechnology Center, University of Wisconsin-Madison, Madison, Wisconsin, United States of America; 4 Laboratory of Genomic Integrity, National Institute of Child Health and Human Development, National Institutes of Health, Bethesda, Maryland, United States of America; Université Paris Descartes, INSERM U1001, France

## Abstract

DNA polymerase V (pol V) of *Escherichia coli* is a translesion DNA polymerase responsible for most of the mutagenesis observed during the SOS response. Pol V is activated by transfer of a RecA subunit from the 3'-proximal end of a RecA nucleoprotein filament to form a functional complex called DNA polymerase V Mutasome (pol V Mut). We identify a RecA surface, defined by residues 112-117, that either directly interacts with or is in very close proximity to amino acid residues on two distinct surfaces of the UmuC subunit of pol V. One of these surfaces is uniquely prominent in the active pol V Mut. Several conformational states are populated in the inactive and active complexes of RecA with pol V. The RecA D112R and RecA D112R N113R double mutant proteins exhibit successively reduced capacity for pol V activation. The double mutant RecA is specifically defective in the ATP binding step of the activation pathway. Unlike the classic non-mutable RecA S117F (*recA1730*), the RecA D112R N113R variant exhibits no defect in filament formation on DNA and promotes all other RecA activities efficiently. An important pol V activation surface of RecA protein is thus centered in a region encompassing amino acid residues 112, 113, and 117, a surface exposed at the 3'-proximal end of a RecA filament. The same RecA surface is not utilized in the RecA activation of the homologous and highly mutagenic RumA'_2_B polymerase encoded by the integrating-conjugative element (ICE) R391, indicating a lack of structural conservation between the two systems. The RecA D112R N113R protein represents a new separation of function mutant, proficient in all RecA functions except SOS mutagenesis.

## Author Summary

DNA polymerase V from the bacterium *Escherichia coli* is one of a class of DNA polymerases that replicate DNA inaccurately. They thus generate mutations at elevated levels. Whereas this might seem incongruous with the goal of accurate transmission of genetic information from one generation to the next, it is actually part of a specialized bacterial DNA repair process that comes into play principally when cells are severely stressed by high loads of DNA damage. Polymerase V is normally inactive. The transfer of one subunit from the bacterial recombinase RecA, and the addition of ATP, leads to the formation of the active pol V Mutasome (Mut) (UmuD′_2_C-RecA-ATP). The current study delves deeper into this process, initiating the task of mapping out the molecular details of the interaction between RecA and UmuD′_2_C. One surface region on the RecA protein required for this activation is defined, the step in the activation process that is affected by this surface is identified, and a direct interaction (or at least very close proximity) between this surface and particular amino acid residues in the UmuC protein is demonstrated. A new RecA variant protein is generated that provides improved separation of function, in that the activation of pol V is abolished while minimally affecting other RecA functions. The study also provides a molecular consummation for a series of incisive genetic studies carried out nearly two decades ago.

## Introduction

The *Escherichia coli* RecA protein is the prototype of a bacterial recombinase common to every species of free-living bacteria [1–3]. Homologs of RecA protein exist in all classes of organisms, where they play central roles in homologous genetic recombination and recombinational DNA repair. RecA forms extended nucleoprotein filaments on single-stranded DNA (ssDNA), and these filaments play a critical role in all RecA functions. *E*. *coli* RecA is a DNA-dependent ATPase. On ssDNA, the RecA filaments have a polarity, and tend to assemble and disassemble by the addition of subunits on the 3' end and subtraction of subunits (after ATP hydrolysis) from the 5' end [4–10].

In *E*. *coli*, RecA has several functions. First, it is a recombinase, responsible for aligning homologous DNA molecules and promoting a strand exchange between them. In this capacity, it has a primary role in the repair of stalled replication forks, as well as for the recombination associated with conjugation and transduction [1–3]. Second, RecA has a regulatory function in the induction of the bacterial DNA damage SOS response. RecA acts as a co-protease, stimulating the autocatalytic cleavage of the LexA repressor that regulates genes of the SOS regulon [11–13], and autocatalytic cleavage of UmuD protein producing the mutagenically active UmuD' [14–16]. Finally, RecA protein activates DNA polymerase V (pol V) [17]. In the context of work carried out on RecA function in SOS mutagenesis, and particularly on activation of pol V (as here), active RecA filaments formed on ssDNA have classically been referred to as RecA*.

The SOS response to DNA damage involves a staged activation of more than 40 genes, and occurs in at least two phases [18–25]. In the early phase, the induced proteins promote relatively accurate DNA repair processes, including recombinational DNA repair and excision repair. If DNA damage is so extensive that these repair functions fail, the cell turns to error-prone translesion DNA synthesis pathways. In this late SOS stage, specialized mutagenic DNA polymerases (II, IV, and V) are induced that are capable of replicating past DNA lesions, a process called translesion synthesis or TLS [17,26–37]. DNA polymerases II and IV are induced from the constitutive levels normally present in cells. DNA polymerase V (pol V) is unique in its tight regulation and general absence except during the SOS response. High levels of potentially deleterious mutagenesis result, but this act of biological desperation allows the survival of at least some cells.

*E*. *coli* pol V is one of the TLS polymerases induced in the late stage of SOS, and is responsible for most of the mutagenesis associated with the SOS response [21,29,35,38–41]. Pol V is derived from the SOS-induced proteins UmuC and UmuD, and is a heterotrimeric complex of UmuD′_2_C [42,43] that has little activity on its own [43–45]. RecA* activates pol V by transferring an ATP-bound RecA subunit from the 3′-proximal end to form the active pol V Mutasome (Mut) (UmuD′_2_C-RecA-ATP)[17,33]. Pol V Mut has an intrinsic DNA-dependent ATPase activity [46]. ATP is required to bind primer-template DNA and ATP hydrolysis triggers dissociation from DNA [46]. The molecular pathway of this process has been described in increasing molecular detail [17,33,46]. However, little information is available about the relevant protein-protein interactions between RecA and UmuD′_2_C.

Enzymes closely related to pol V are encoded by naturally occurring R plasmids and conjugative elements, including the MucA′_2_B polymerase from the incN plasmid R46 and the RumA′_2_B polymerase encoded by the ICE R391 [47–51]. Like UmuD′_2_C, both of these enzymes are activated by RecA*. *In vitro*, the activated MucA′_2_B enzyme is more proficient in the bypass of lesions than is pol V Mut, although it bypasses T-T *cis-syn* cyclobutane dimers more accurately than does pol V Mut [50]. RumA′_2_B on the other hand, promotes higher levels of spontaneous mutagenesis *in vivo* compared to both UmuD′_2_C and MucA′_2_B [51]. Like MucA′_2_B, RumA′_2_B is two-three fold more accurate than UmuD′_2_C at bypassing T-T *cis-syn* cyclobutane dimers *in vitro* [51] and *in vivo* [52].

Important clues to defining at least some of the RecA surface residues important for pol V activation came from the work of Devoret, Sommer, Bailone, and colleagues [53–59]. At a time pre-dating the discovery of pol V, these workers first observed that over-expression of the UmuD and UmuC proteins at unnaturally high levels *in vivo* results in suppression of RecA mediated recombination processes [54,60]. A similar phenomenon was noted with the MucAB proteins [49]. Inhibition of RecA protein by UmuDC has also been observed *in vitro* [61]. RecA mutants were subsequently isolated that exhibited resistance to the UmuDC inhibition, called Umu^R^ mutations (Umu resistant) [57]. Most of these mutations clustered on the RecA protein surface defined by residues D112, N113, L114, and S117. Additional RecA mutations with similar properties were isolated at residues S44 and V247 [57]. The S44 residue occurs at a subunit-subunit interface, and may alter the filament to make it more resistant to high levels of UmuD′_2_C [57]. Residues S44 and V247 have not been further studied.

Of the RecA surface residues, a mutation at position 117 (S117F), has been more fully characterized and has been used in numerous studies, primarily as a control in efforts to explore Umu-dependent mutagenesis [53,55,56] and the mechanism of pol V activation [17,33,62–64]. RecA S117F (recA1730) was first isolated as part of a study aimed at genetic separation of the various RecA functions [55]. Notably, the very same allele appeared three times among 11 RecA mutations isolated that were resistant to recombination inhibition by high levels of UmuD and UmuC proteins [57], suggesting that this amino acid residue was involved in a RecA-Umu interaction. RecA S117F will not activate DNA polymerase V [17,63,64]. RecA S117F retains a limited capacity for recombination and SOS induction *in vivo*, but only when over-expressed [55]. *In vitro*, the RecA S117F protein is deficient in ATP hydrolysis and filament formation on ssDNA is substantially but not completely reduced [56], compromising its status as a complete separation of function mutant.

The function retained by RecA S117F is sufficient to activate high levels of mutagenesis by the DNA polymerase RumA′_2_B [51]. Facile activation of RumA′_2_B but not UmuD′_2_C-mediated mutagenesis *in vivo* by RecA S117F defines these two polymerases as potentially complementary platforms with which to explore critical RecA interaction/activation surfaces.

Some of the same RecA surface residues have subsequently factored into studies of RecA proteins that confer a hyper-recombinogenic phenotype [65]. A mutation at position 112, D112R, was particularly effective at increasing recombination frequency during conjugation [65]. Recognizing the preponderance of Umu^R^ mutations that clustered in this region of the RecA protein [56], we have embarked on a new effort to investigate the effects of alterations in RecA residues 112–117. Here, we report on a separation-of-function RecA double mutant, D112R N113R, which effectively blocks the ATP binding step of pol V activation while minimally affecting other RecA functions. We find that the RecA 3′-exposed surface at residue N113 interacts primarily with at least two surfaces on the UmuC subunit of pol V, indicating the presence of multiple conformational states. The results provide the first definition of interacting RecA and UmuC surfaces critical to pol V activation. We also demonstrate that this same surface on RecA plays less of a role in the activation of RumA′_2_B.

## Results

The goal of this study was to explore the biological and biochemical properties of the RecA protein surface in the region of the amino acid residues 112–117. Past efforts to characterize the properties of the S117F mutant [17,33,53,55,62–64,66], and the presence of RecA alleles that conferred a hyper-recombinogenic phenotype at residues 112 and 113 [65], led us to focus on genetic alteration of positions 112, 113, and 114. A series of RecA mutant proteins were constructed and expressed, including the variants D112R, N113R, and L114R, as well as the double mutant containing D112R and N113R. The arginine substitution was chosen in an attempt to disrupt any potential protein-protein interaction as much as possible, with the understanding that some potential existed to destabilize the RecA protein structure. Indeed, the L114R substitution produced a protein that exhibited little if any RecA function. This RecA variant protein may not fold properly or may not form active nucleoprotein filaments. The other two substitutions generated RecA proteins that were demonstrably active in standard RecA functions. We did not pursue work with the RecA N113R protein individually since early assays *in vivo* indicated only a modest effect on pol V-mediated mutagenesis. Thus, most of the experiments to follow focus on the D112R mutant and the D112R N113R double mutant. A series of experiments were carried out to examine the function of these RecA mutant proteins both *in vivo* and *in vitro*. Incorporation of a photo-reactive unnatural amino acid at N113 allowed us to probe the dynamics of pol V activation in the presence or absence of key substrates necessary for DNA synthesis.

Within an active RecA nucleoprotein filament, the surface defined by residues 112–117 are at or near the subunit-subunit interface. In the structure of a RecA filament bound to ssDNA [67], residues 112 and 113 are closely proximal to residues 28–30 in the adjacent subunits. It is not yet know how these interactions might change due to conformation changes during the ATP hydrolytic cycle and during DNA strand exchange.

### RecA protein with alterations at positions 112 and 113 promote normal UmuD cleavage, SOS induction, and recombination functions *in vivo*

**Fig 1:**
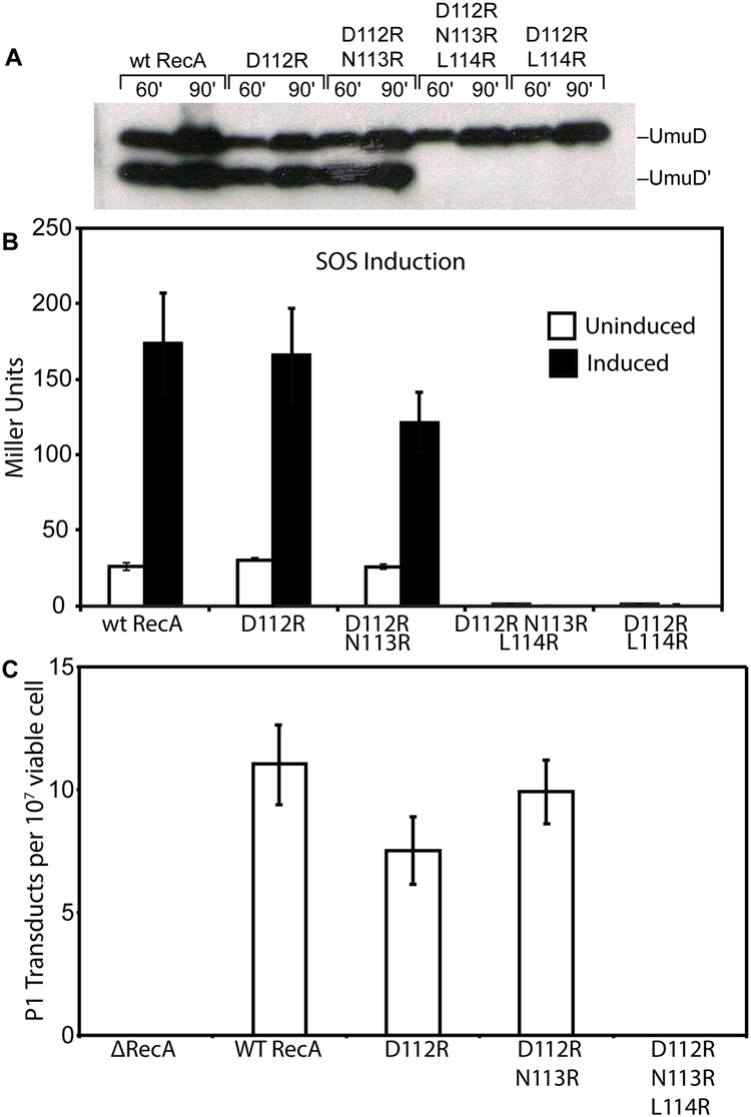
*In vivo* activities of RecA mutants. (A) Cleavage of UmuD *in vivo*. Western blot analysis of UmuD cleavage to UmuD' was done as described in Methods. Strains harboring *recA* mutants on the chromosome where transformed with pJM103 (UmuD) and pT7 pol26 (Tet^R^) in order to visualize UmuD cleavage. Cells were induced for UmuD production and treated with 60 J/m^2^ to induce autocatalytic cleavage by the *recA* mutants. An equal cell OD was loaded for each sample and visualized with anti-UmuD/D' antibodies. (B) Induction of the SOS response *in vivo*. β-galactosidase assays were performed in cells uninduced and induced for the SOS response after treatment with a UV dose of 30 J/m^2^. An SOS reporter plasmid was transformed into strains with the *recA* mutants on the chromosome as described in Methods. Miller units were calculated for each strain. (C) RecA-dependent recombination *in vivo*, as measured by P1 transduction efficiency. Strains harboring the *recA* mutants on the chromosome were transduced with P1 carrying a Kan cassette. Kan^R^ colonies were used to determine efficiency of RecA-dependent homologous recombination.

We examined several key RecA protein functions *in vivo*. For this task, all of the RecA protein variants were expressed on the *E*. *coli* chromosome, at the same location and utilizing the same promoter as the wild-type *recA* gene. As shown in Fig. 1A, both RecA D112R and RecA D112R N113R proteins were proficient in the *in vivo* autocatalytic cleavage of the UmuD protein visualized via Western blot. The variants with the L114R change were completely deficient in UmuD cleavage. The effects of these mutations on SOS induction were determined by measuring the β-galactosidase activity from an SOS (*recN*) promoter. Background SOS response levels were at least as proficient in the presence of the RecA D112R and RecA D112R N113R proteins as was observed for the wild-type protein (Fig. 1B). Cells were also treated with 30 J/m^2^ UV to induce a strong SOS response. The D112R was comparable to the wild-type protein, while the D112R N113R tended to produce somewhat lower levels that were still within error of the wild-type protein. Finally, recombination function was examined utilizing an assay based on bacteriophage P1 transduction (Fig. 1C). Again, the RecA D112R and RecA D112R N113R mutant proteins were proficient, although the D112R variant generated somewhat fewer transductants. We note that this same D112R variant generates a much higher level of recombinants in a conjugation-based assay than does the wild-type protein [65]. In all of these assays, constructs that also included the L114R mutation were inactive. Therefore, strains carrying this mutation were not studied further, due to non-functional RecA activity and likely deficiency in filament formation.

### RecA D112R N113R protein is deficient in SOS mutagenesis

**Fig 2:**
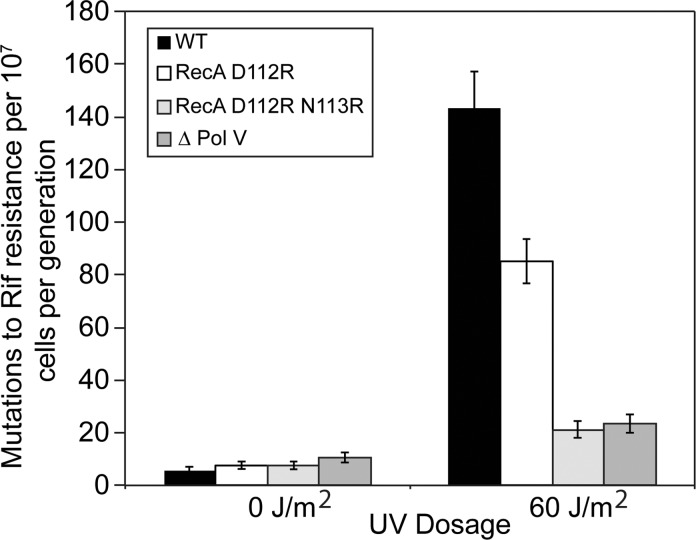
The RecA D112R N113R variant reduces SOS mutagenesis to levels comparable to strains lacking DNA polymerase V. The majority of rifampicin resistant colonies are dependent upon pol V SOS mutagenesis. A rifampicin mutation assay was used to determine mutation rate from 15-tube fluctuation analyses, and 95% confidence limits were calculated as described in Methods. All strains were derived from MG1655. Briefly, cells were grown to mid-log and exposed to 0 J/m^2^ or 60 J/m^2^ to induce SOS, grown overnight at 37°C, and plated on Rifampicin and LB plates. Colony counts were performed followed by fluctuation analysis.

We examined the ability of mutant RecA proteins to activate pol V *in vivo*, which can be followed via SOS mutagenesis. Utilizing a mutagenesis assay based on the production of rifampicin-resistant variants of RNA polymerase after UV irradiation, the level of mutagenesis was reduced almost 50% when the *recA* D112R variant replaced the wild-type *recA* (Fig. 2). The *recA* D112R N113R strain had SOS mutagenesis levels further reduced to those seen in a strain lacking pol V (Fig. 2). In order for SOS mutagenesis to occur, a functional RecA is necessary to proceed through a series of regulatory processes. The abolished SOS mutagenesis seen with *recA* D112R N113R reflects defective activation of pol V by this variant. However, it could also be due to any of the following: deficient SOS induction (no expression of UmuC/UmuD), lack of UmuD autocatalytic cleavage (no active UmuD′), and lastly irregular RecA filament formation essential for activation of pol V. In order to probe this lack of SOS mutagenesis further, the RecA D112R N113R protein was purified.

### RecA protein with alterations at positions 112 and 113 are proficient in all *in vitro* functions of RecA protein

**Fig 3:**
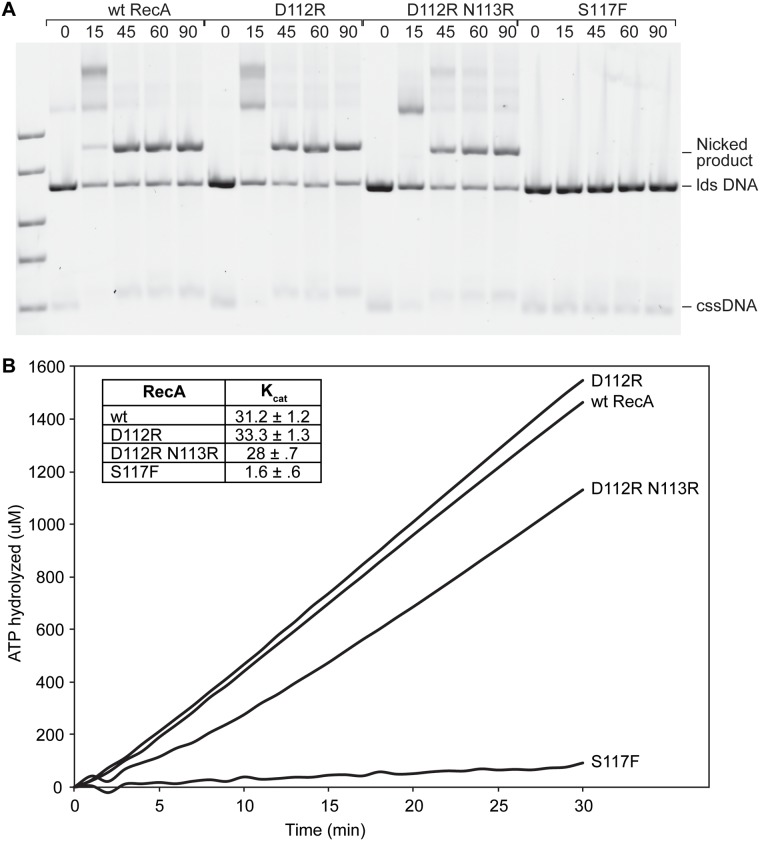
RecA D112R and RecA D112R N113R are capable of strand exchange and ATP hydrolysis. (A) Reactions were carried out as described in Methods. The reaction solutions contained 10 μM M13mp18 cssDNA, 3.5 μM RecA, 20 μM M13mp18 ldsDNA, 3 mM ATP, and 1 μM SSB in standard RecA buffer. RecA forms filaments on cssDNA, catalyzes pairing with the homologous ldsDNA, and upon completion of strand exchange a nicked circular product is formed. At least four repeats with the wt RecA, D112R, D112R N113R proteins were conducted. Two of these repeats also included trials with the RecA S117F protein, and a representative gel is shown. (B) The RecA-catalyzed ATP hydrolysis in the presence of cssDNA was monitored over time. The reactions were performed as described in Methods. The RecA D112R and D112R N113R exhibit normal ATPase rates, while the S117F minimally hydrolyzes ATP. Time zero corresponds to the time of ATP/SSB addition.

The RecA D112R and RecA D112R N113R variants were studied *in vitro*. The classical RecA S117F that fails to activate pol V was also included in many trials for comparison. Both RecA D112R and RecA D112R N113R proteins are proficient in the catalysis of a standard RecA-mediated DNA strand exchange reaction, as shown in Fig. 3A. In this reaction, full length circular ssDNA and linear dsDNA substrates derived from bacteriophage M13mp18 are converted by RecA to a circular nicked dsDNA product. The product band is indicated in Fig. 3A. Levels of product were generally comparable to those seen with the wild-type protein. The RecA S117F is completely deficient in the strand exchange reaction when tested under similar conditions. The experiments with the wild-type, D112R, and D112R N113R RecA variants were all repeated four times or more with consistent results. The experiments with RecA S117F was carried out twice, in each case showing no detectable generation of strand exchange products.

Since RecA* is a DNA-dependent ATPase, DNA binding can be indirectly measured using ATP hydrolysis. The rates of ATP hydrolysis observed for RecA D112R and RecA D112R N113R bound to circular ssDNA were 33.3 ± 1.3 μM/min and 28.0 μM/min, respectively (Fig. 3B). The slight decrease in ATP hydrolytic activity by RecA D112R N113R was highly reproducible. The RecA S117F failed to show normal ATP hydrolysis rates (Fig. 3B) under similar conditions, indicating a substantial deficiency in single-stranded DNA binding and/or ATP hydrolysis activity.

**Fig 4:**
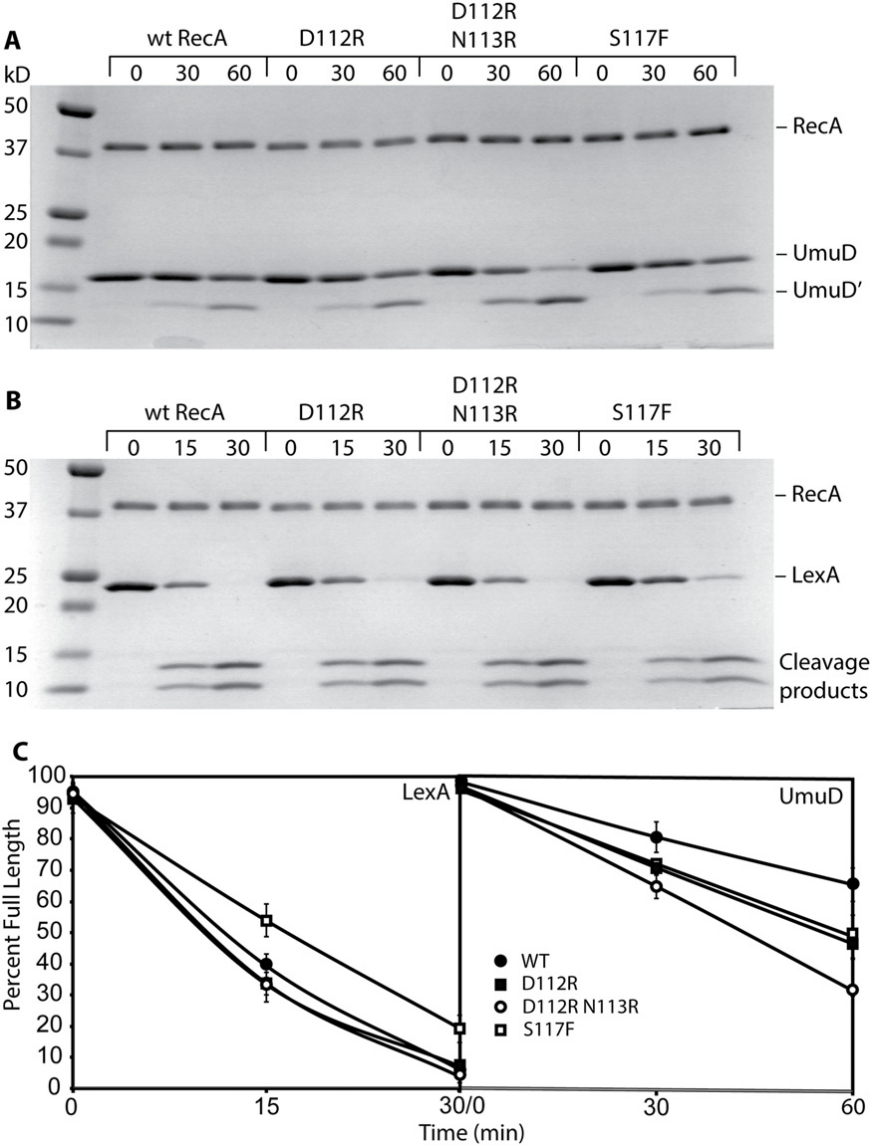
RecA D112R and RecA D112R N113R promote autocatalytic cleavage of LexA and UmuD *in vitro*. Reactions were carried out as described in Methods. At least four repeats with all RecA variants were conducted. Representative gels are shown. (A) Cleavage of UmuD protein. Reactions containing 5 μM of M13mp18 cssDNA, 3 μM RecA, 3 mM ATPγS, and 8 μM UmuD were incubated at 37°C for 0, 15, and 60 minutes and visualized by staining with Coomassie blue. (B) Cleavage of LexA protein. Reactions containing 5 μM of M13mp18 cssDNA, 3 μM RecA, 3 mM ATPγS, and 8 μM LexA were incubated at 37°C for 0, 15, and 30 minutes. The products of the reaction were separated on a 15% SDS-PAGE and visualized as in (A). (C) Quantification of the LexA and UmuD *in vitro* cleavage.

Both RecA D112R and RecA D112R N113R were very comparable to the wild-type protein in their capacity to promote the autocatalytic cleavage of both the UmuD and LexA proteins (Fig. 4A-C). The S117F was somewhat more robust than wild-type RecA in UmuD cleavage, but was slightly less robust in the LexA cleavage reaction (Fig. 4A, 4B). The gapped or incomplete RecA filaments formed by RecA S117F [56] are sufficient to support these autocatalytic cleavage events.

Overall, these data provide evidence that the RecA D112R and RecA D112R N113R, in contrast to RecA S117F, are fully functional in regards to standard RecA activities, including filament formation, ATP hydrolysis, and DNA strand exchange. RecA S117F does form filaments that are sufficient to support LexA and UmuD autocatalytic cleavage. Therefore, the loss of SOS mutagenesis *in vivo* suggests that these variants specifically lack the capacity for pol V activation.

### The RecA D112R N113R variant is defective in the activation of DNA polymerase V *in vitro*

**Fig 5:**
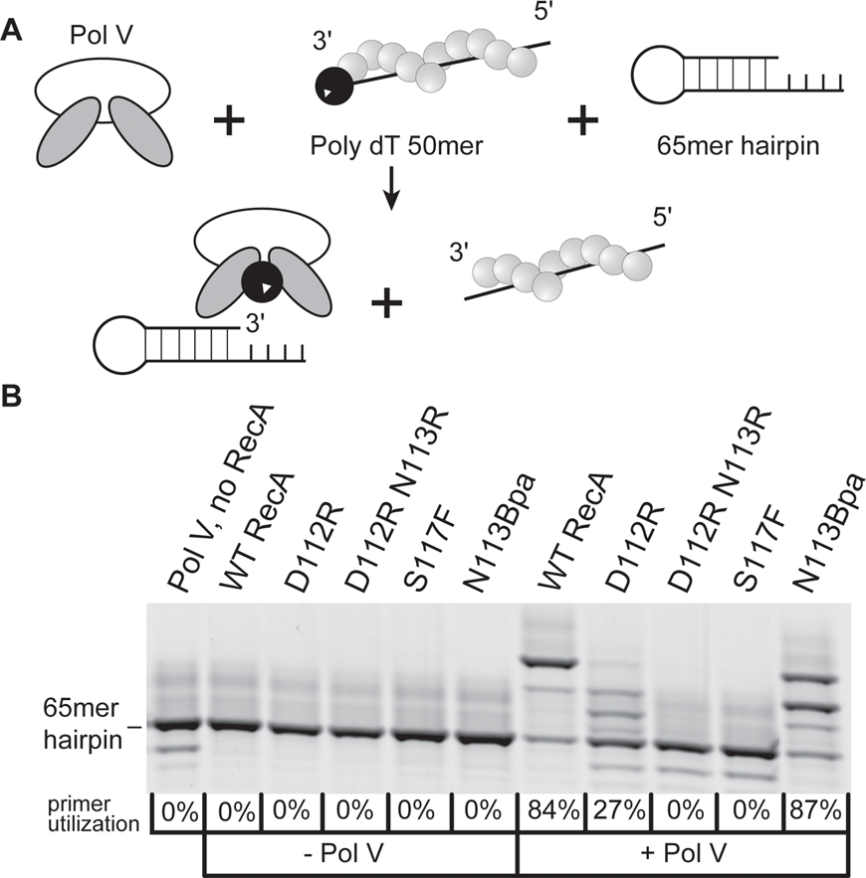
RecA variant D112R N113R is defective in DNA polymerase V activation. (A) Schematic of DNA synthesis by pol V transactivated by RecA*. (B) In pol V reaction buffer, 5 mM DTT, 2 mM ATPγS, 2 μM poly (dT)_50_, and 20 μM RecA were incubated at 37°C for 10 minutes to allow for formation of RecA*. This was followed by the addition of 1 mM dNTPs, 1 μM pol V, and 110 nM p/t hairpin DNA and incubation for 1 hour at 37°C. During this time pol V Mut is formed, synthesizes DNA, and can be reactivated for another round of synthesis (right panel, +Pol V). Reactions were repeated without the addition of pol V to demonstrate the absence of contaminating polymerase in the RecA preps (left panel,—Pol V).

The capacity of RecA variants to activate pol V was assayed using an established pol V transactivation assay [64]. Inactive pol V (UmuD′_2_C) is activated by RecA* formed on poly dT DNA (Fig. 5A). The activated pol V Mut (UmuD′_2_C—RecA—ATP) is then capable of synthesis on a hairpin primer-template DNA. The RecA* filaments remained in the solution throughout the experiments and are capable of reactivating pol V. The 4-nucleotide overhang on the hairpin substrate is too short to support RecA filament formation. In the presence of only RecA proteins or pol V, no primer utilization is observed. The wild-type RecA protein robustly activated pol V with 84% primer utilization as previously seen [64] (Fig. 5B). In the presence of RecA D112R, there is only 27% primer utilization. For the RecA D112R N113R variant, primer utilization was not detectable. As previously shown, the RecA S117F does not activate pol V [17,63,64]. In this assay, the RecA D112R N113R protein is as defective as RecA S117F in activation of pol V (in this assay), while retaining all other RecA functions (Fig. 5B). There is no DNA synthesis observed above background when pol V is omitted, which shows that none of the other *E*. *coli* polymerases are present as contaminants in the reaction (Fig. 5B).

**Fig 6:**
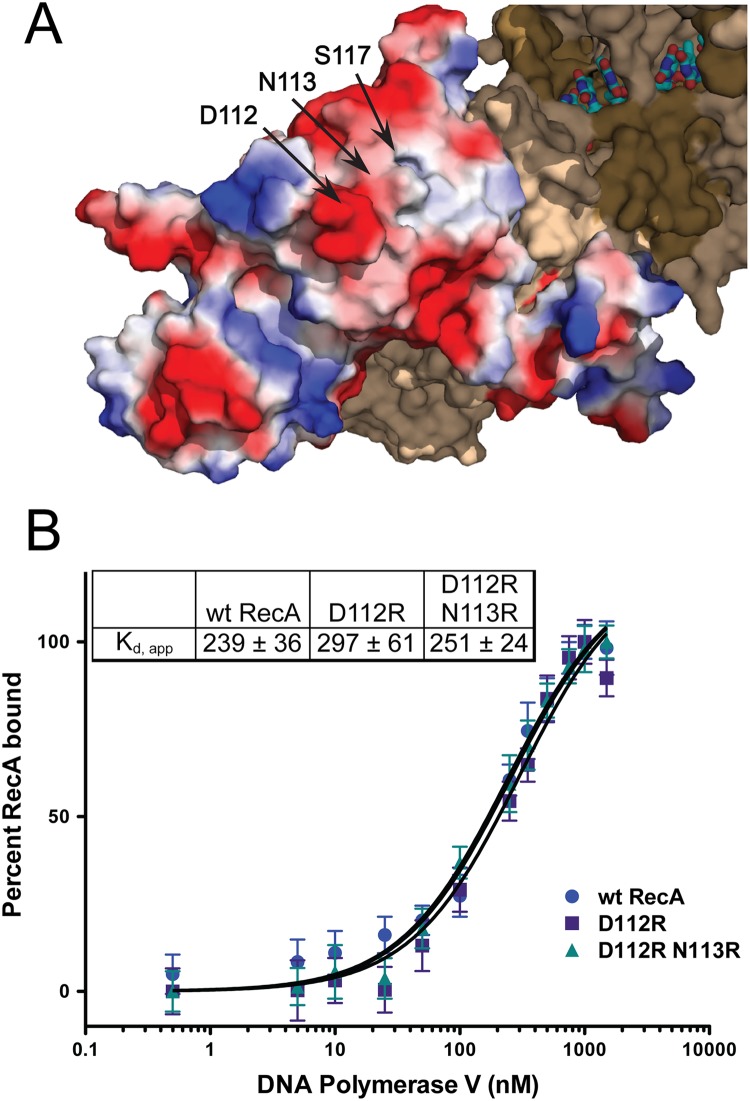
RecA D112R and D112R N113R exhibit wild-type RecA binding affinities for pol V. (A) Location of investigated residues on the RecA protein surface. The D112 and N113 residues compose an acidic knob on the RecA surface. The RecA monomer represented in electrostatic coloring scheme (red = negative charged residues, blue = positive charged residues) was generated in Pymol (PDB 3CMU [67]). In this illustration, the monomer shown is located at the 3' end of the ssDNA (the 3'-proximal RecA monomer), which is the monomer removed by pol V during the activation cycle. (B) Altering this acidic surface to basic residues does not affect the binding affinity for pol V. Equilibrium binding isotherms of wild-type RecA, RecA D112R, and RecA D112R titrated with pol V as monitored by fluorescence depolarization. All data are the average of at least three experiments. Error bars are one standard deviation from the mean.

Upon analysis of the 3'-exposed surface of RecA, residues 112, 113, and 117 seemed to cluster in or near an acidic knob and hydrophobic area (Fig. 6A). We first hypothesized that this lack of activation was a result of a disrupted RecA-pol V interaction. The wild-type RecA, RecA D112R, and RecA D112R N113R were each labeled with fluorescein to investigate their interactions with pol V (UmuD′_2_C) in solution as previously described [63]. It should be noted that simply mixing RecA protein with UmuD′_2_C (as in this experiment) leads to formation of a complex, but this complex is inactive in translesion DNA synthesis [63]. Similar to RecA S117F [63], the RecA D112R and RecA D112R N113R exhibited binding affinities (K_d_,_app_ ~250 nM) comparable to wild-type RecA (Fig. 6B). Therefore, the deficiency in activation of pol V by RecA D112R and RecA D112R N113R does not reflect a complete disruption of pol V-RecA interaction. The interaction of the RecA variants with UmuD′_2_C in the active pol V Mut is described in experiments presented below.

### Mutations at positions 112, 113, and 117 do not abolish RecA-mediated activation of RumA′_2_B

**Fig 7:**
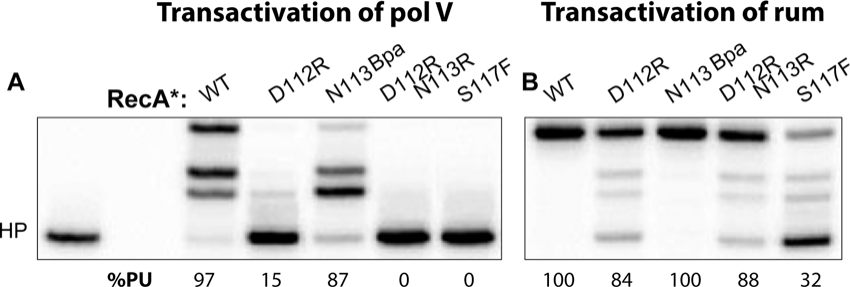
RumA′_2_B can be activated by all RecA variants. Transactivation reaction was carried out on 3-nt overhang hairpin for UmuD′_2_C (A) and RumA′_2_B (B). RecA* (400 nM) was preformed on 30 nt ssDNA then mixed with UmuD′_2_C or RumA′_2_B (400 nM each) and incubated for 30 mins at 37°C. Unlike UmuD′_2_C (A), RumA′_2_B can be activated by RecA variants D112R, D112R N113R, and S117F (B).

The RecA protein also plays an important and similar role in the activation of the pol V-related polymerase RumA′_2_B. We wished to determine if the molecular interactions involved in activation were conserved in the RumA′_2_B system. Another transactivation assay employing a 3-nt overhang hairpin primer-template was carried out to explore the activation of RumA′_2_B (Fig. 7). As also seen in Fig. 5, neither RecA D112R N113R nor RecA S117F activated UmuD′_2_C (Fig. 7). In this assay, the RecA D112R protein was also partially defective in activation. Consistent with previous *in vivo* results [47,48,51], the RecA S117F mutant activated the RumA′_2_B polymerase *in vitro* (Fig. 7). The RecA D112R and RecA D112R N113R mutant proteins also activated RumA′_2_B polymerase. In general, activation of RumA′_2_B resulted in more robust polymerase function than did activation of UmuD′_2_C, again consistent with the robust levels of mutagenesis seen with this enzyme *in vivo* [47,48]. As with the LexA and UmuD autocatalytic cleavage assays, the RecA S117F protein retains sufficient function to activate a polymerase closely related to pol V, although the activation is somewhat less proficient than observed for wild-type RecA or the RecA D112R N113R mutant (Fig. 7). This indicates that the inability to activate DNA polymerase V is not due to its deficiencies in RecA filament formation. The results also indicate that the RecA surface centered on residues 112 and 113 is not involved in activation of RumA′_2_B, even though it is critical for activation of UmuD′_2_C. Overall, the results indicate that the RecA-pol interactions involved in activation of UmuD′_2_C are at least partially distinct from those involved in activation of RumA′_2_B.

### Activation of UmuD′_2_C by RecA D112R N113R is blocked at the ATP binding step

**Fig 8:**
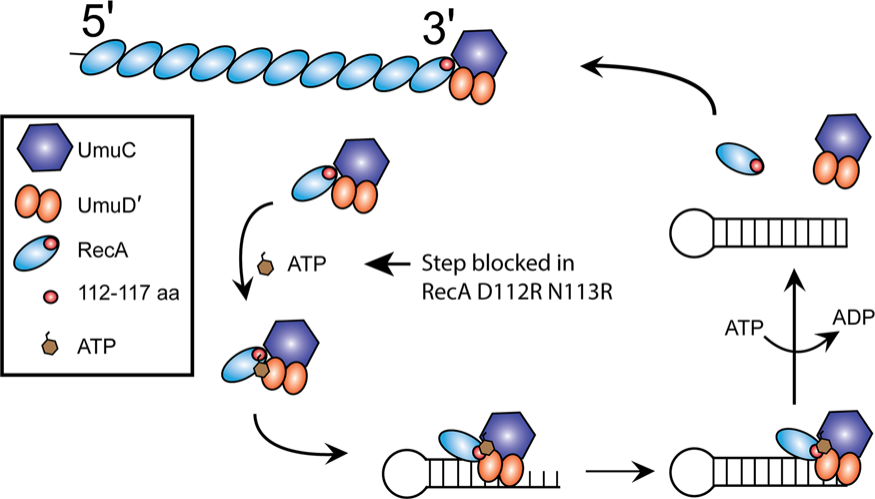
Model for pol V activation. A 3'-RecA monomer is transferred to pol V, to form pol V Mut which is unable to bind to primer-template DNA. The RecA 112–117 aa are close to UmuC in this initial interaction. ATP is bound to the complex to complete the activation, followed by binding to the primed template. We hypothesize that conformational changes alter the interaction between the RecA subunit and the UmuD′_2_C subunits at successive stages of the reaction, and may make transient contacts with UmuD'. Pol V Mut will synthesize on the template, and can only dissociate from the primer-template DNA upon ATP hydrolysis. By exchanging the RecA subunit in the inactive complex for a new subunit obtained from RecA*, this released pol V can then be reactivated for another round of synthesis if ssDNA and RecA* are still present.

The pathway by which UmuD′_2_C is activated by RecA protein first involves the removal of a RecA subunit from the 3'-proximal end of a RecA* filament (Fig. 8). ATP is then bound, and the activated UmuD′_2_C-RecA-ATP complex then binds to a primed template [17,33,46]. Association of this complex to the β-clamp is also a feature of this process *in vivo* [39,68–74], although this aspect of the reaction is not explored here.

**Fig 9:**
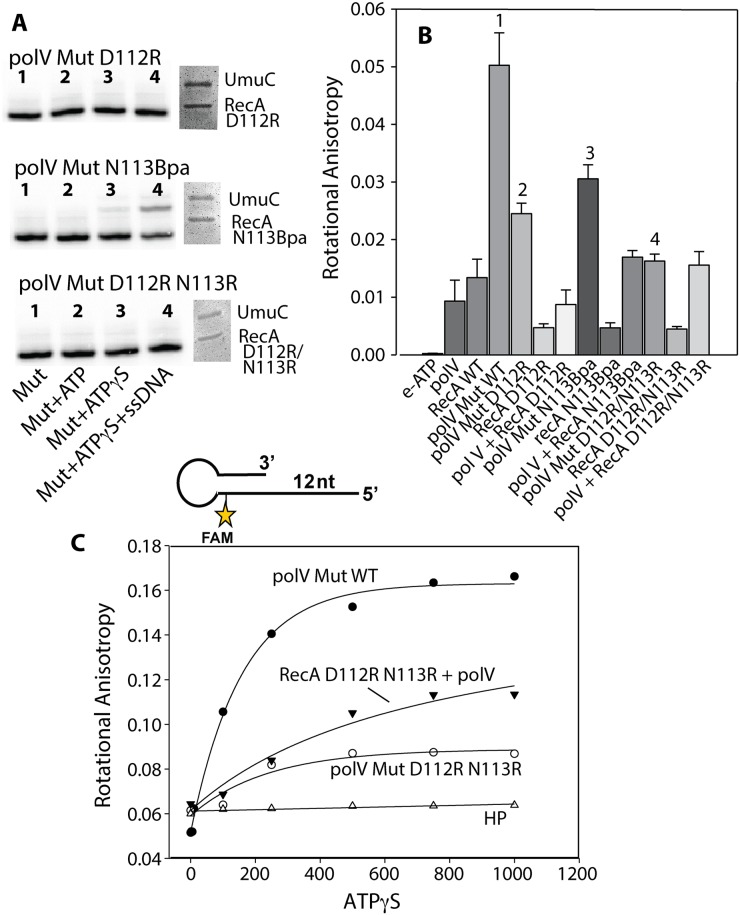
Defining the step in the pol V activation pathway that is affected by RecA D112R N113R. Active DNA polymerase V-Mut was generated with multiple RecA variants as described in Methods, and subjected to several experimental procedures. (A) The activity of pol V Mut D112R, pol V Mut N113RBpa and pol V Mut D112R N113R (400 nM each) was detected on 5'-^32^P-labeled 12 nt oh HP (100 nM) in the presence or absence of ATP/ATPγS and dNTPs. ssDNA and ATPγS were added to the reactions to detect free RecA and determine whether pol V Mut could be transactivated (left panels). Pol V Mut variants were resolved by SDS-PAGE and protein components were visualized via Imperial staining (right panels). (B) Binding of pol V Mut and pol + RecA mixtures (400 nM each) to ε-ATP (500 μM) was measured using rotational anisotropy. The error bars correspond to 1 SEM. Numbers above four of the bars are described in the text. (C) Binding of pol V Mut WT (400 nM), pol V + RecA D112R N113R (400 nM each) and pol V Mut D112R N113R (400 nM) to fluorescein-labeled 12 nt oh HP (50 nM) was measured as a function of ATPγS. DNA binding was observed as a change in rotational anisotropy. The rotational anisotropy of HP alone is included in the graph.

The anisotropy results of Fig. 6 indicate that RecA D112R and RecA D112R N113R are still able to interact with pol V with similar affinity as RecA WT. However, the protocol in those experiments does not generate the active complex, pol V Mut. Although RecA D112R and RecA D112R N113R do not transactivate pol V, they may be able to from a pol V Mut complex. We used RecA D112R, RecA with a residue of *p*-benzoyl-phenylalanine (Bpa) substituted for Asn at position 113 (N113Bpa) and RecA D112R N113R to assemble pol V Mut, and resolved the flow-through collected from the spin columns (see Materials and methods) via SDS-PAGE to determine the components. A clear UmuC and RecA band can be observed for all pol V Mut variants, suggesting that pol V is able to strip a RecA monomer from the 3' tip of RecA* formed with all of the RecA mutants (Fig. 9A). The protein gels indicate that the ability to form a pol V Mut-like complex with the RecA variants does not seem to be compromised in any of the mutants. However, pol V Mut formed with RecA* D112R and pol V Mut D112R N113R are not active for DNA synthesis while pol V Mut N113Bpa displays some activity (Fig. 9A).

It has been shown that pol V Mut WT binds ε-ATP in the absence of p/t DNA, whereas equimolar concentrations of pol V and RecA alone do not [46], providing an indirect method to determine whether pol V and RecA are in a complex. Pol V Mut D112R has decreased binding to ε-ATP compared to pol V Mut WT (Fig. 9B; bar #2 vs. bar #1), likely explaining the lack of DNA synthesis (Figs. 9A and 5B). However, the pol V Mut D112R result can be distinguished from that seen with pol V + RecA D112R binding to ε-ATP (to form the inactive complex) suggesting the presence of an enzyme complex when pol V Mut D112R is formed. A decrease in rotational anisotropy was also observed for pol V Mut N113Bpa; however, the decline was less than observed for the other pol V Mut variants (Fig. 9B; bar #3), supporting the reduced but still observable extension of p/t DNA for this variant (Fig. 9A). Surprisingly, binding to ε-ATP was greatly diminished for pol V Mut D112R N113R, displaying similar levels of ε-ATP as in the inactive complex formed by simply mixing pol V + RecA D112R N113R (Fig. 9B; bar #4).

If pol V Mut D112R N113R is a complex as implied by the SDS-PAGE results (Fig. 9A), the inability to bind ATP efficiently should be reflected in a lack of binding to p/t DNA. To this end we measured the ability of pol V Mut D112R N113R to bind 12 nt oh HP as a function of ATPγS concentration (Fig. 9C). Pol V Mut WT, which efficiently binds ε-ATP, also binds p/t DNA. Binding of pol V Mut D112R N113R to HP DNA is greatly diminished, resulting in a lower rotational anisotropy compared to an inactive mixture of pol V + RecA D112R N113R (Fig. 9C). Since RecA D112R N113R retains most of its RecA functions, including binding to DNA, the change in rotational anisotropy observed when pol V and RecA D112R N113R are mixed is likely due to DNA binding by small amounts of the free form of the RecA variant protein. In general, the results in Fig. 9 indicate that activation of pol V by RecA D112R N113R is most likely blocked at the stage of ATP binding.

### The RecA with *p*-benzoyl-phenylalanine incorporated at position 113 cross-links to amino acid residues on two surfaces of the UmuC subunit of pol V

To explore the possibility of an interaction between this RecA surface and pol V, the photo-cross-linking technique developed by Schultz and his collaborators [75–78] was utilized. This technique enables the introduction of *p*-benzoyl-phenylalanine (Bpa), a photo-reactive phenylalanine derivative, at desired amino acid positions. We expressed RecA N113 with an amber suppressor, tyrosyl tRNA, and tyrosyl tRNA synthetase, both of which are engineered to incorporate Bpa into an amber (TAG) codon. The carbonyl oxygen at the benzophenone group of Bpa preferentially reacts with nearby carbon-hydrogen bonds when irradiated with UV light.

Position 113 places the Bpa at the 3'-exposed surface of RecA (see Fig. 6A), and allowed us to probe the interaction with pol V. Specifically, the Bpa was incorporated at position N113 to help confirm that this surface is involved in activation and determine if this surface interacts with the UmuC or the UmuD' subunits of pol V. The codon at this position was replaced with a TAG stop codon. The RecA N113TAG construct was transformed into bacteria carrying a Bpa-specific suppressor tRNA and aminoacyl-tRNA synthetase, which allowed incorporation of Bpa in place of the TAG stop codon. The RecA N113 construct was expressed in the presence of Bpa and purified. Subsequent MS analysis confirmed the identity of the protein and incorporation of the Bpa at the expected position. As shown in Figs. 5 and 7, the RecA protein substituted with Bpa at position 113 was nearly as proficient in the activation of pol V as was wild-type RecA protein. A difference in the pattern of products formed (Fig. 5B) might reflect some effect of the 113Bpa substitution on the inherent pol V Mut ATPase, which controls processivity [46].

**Fig 10:**
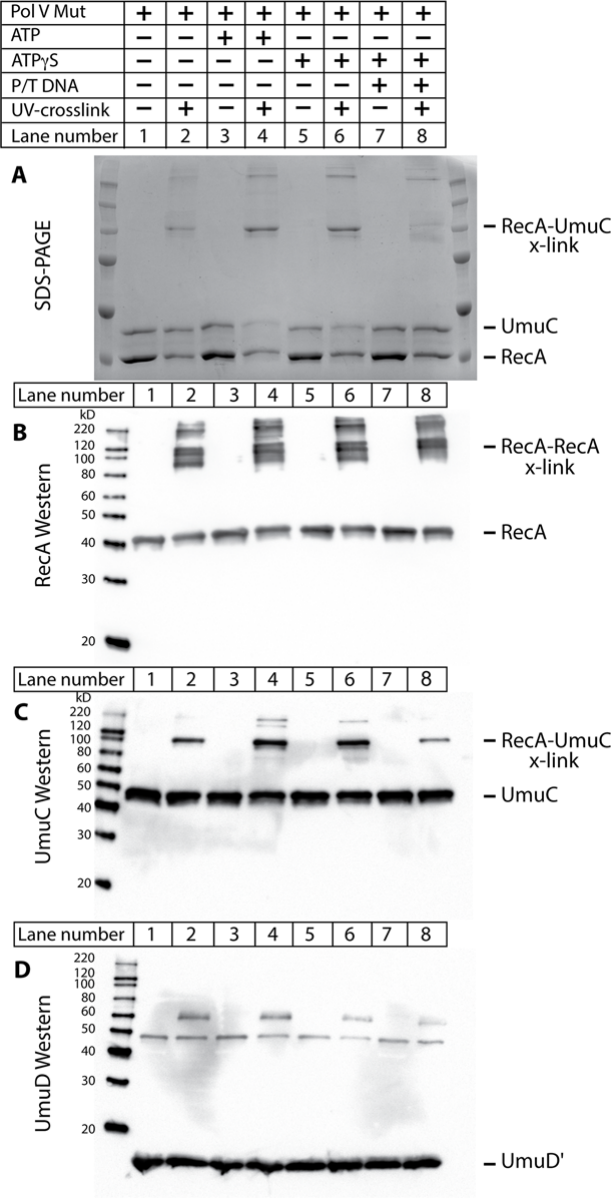
The RecA 3'-surface interacts with UmuC in pol V Mut. The photo-reactive unnatural amino acid Bpa was incorporated into RecA at position N113 (RecA N113Bpa) and used to probe RecA/pol V Mut interactions. The protocol for the generation of pol V Mut was followed as described in Methods, with ATP, ATPγS, and template-primer added where indicated. Samples were exposed to UV light to covalently crosslink the RecA N113Bpa to nearby interacting partners (A) Coomassie stained SDS-PAGE of samples. UmuC, RecA, and UmuD' bands are indicated. Higher molecular weight bands appear upon cross-linking via UV light. The major cross-linked species runs at ~100 kDa. (B) Western blot of reactions presented in (A) using antibodies to the RecA protein. (C) UmuC western blot of reactions presented in (A). UmuC is present in the major cross-linked band at ~100 kDa. (D) UmuD' western blot of reactions presented in (A). A non-specific band is present at ~49 kDa which is not UV crosslinking dependent. A weak band observed at ~60 kDa was UV-dependent, but was not observed via SDS-PAGE. Therefore, it could not be pursued via mass spectrometry analysis.

Two different protocols were used to generate the cross-linked species in the active and inactive complexes of RecA with pol V. In one, pol V-Mut was formed by incubating RecA* tethered to streptavidin beads with UmuD′_2_C, and then removing the tethered RecA* by centrifugation as described in Methods. Once pol V-Mut was formed, ATP, ATPγS, or ATPγS with primer-template DNA were added to the pol V-Mut complex. These complexes are capable of translesion DNA synthesis and are referred to as active complexes. In the second protocol, RecA protein was simply incubated with UmuD′_2_C in the absence of ssDNA, again with ATP, ATPγS, or primer-template DNA. These complexes were inactive in translesion DNA synthesis whether the ATP/ATPγS was added or not, and are referred to as inactive complexes. Cross-linking was induced by irradiation with UV light. The samples generated by the protocol for generating active pol V Mut, along with un-irradiated control samples, were separated by SDS-PAGE and Western blotting was performed to visualize molecular species in the cross-linked bands (Fig. 10). The SDS-PAGE clearly shows a higher molecular weight species (~100 kDa) present in the cross-linked samples, but not in the un-cross-linked samples (Fig. 10A). The composition of this band was analyzed by blotting with α-RecA, α-UmuC and α-UmuD/D' antibodies (Fig. 10B-D). The 100 kDa band is recognized with UmuC and RecA antibodies but not UmuD/D' antibodies. However, the sensitive Western blots did detect species in which RecA was cross-linked either to other RecA molecules or to UmuD' that were not readily observed in the Coomassie-stained gel in panel A. The RecA antibodies detected several multimers of RecA, as well as the 100 kDa species. The band most prominent in the Coomassie-stained gel likely corresponds to one RecA, one UmuC (~85 kDa) being cross-linked, which is consistent with previous studies [17,33,64]. The slight discrepancy in size is likely due to the structure of the cross-linked proteins. To verify molecular composition the bands of interest were excised from the gel, digested with trypsin and AspN proteases, and submitted to LC-MS/MS analysis. Greater than 60% peptide coverage for RecA and UmuC verified these two proteins were present in the 100 kDa cross-linked band. These cross-linked bands of these samples were also analyzed by mass spectrometry in hopes of identifying unique surfaces on UmuC in the active and inactive complexes.

The cross-linked band is present in the absence of ATP or ATPγS (lane 2), as well as when ATP, ATPγS, and/or primer-template DNA is added to pol V Mut. Under the conditions tested, very limited cross-linking to UmuD' was also observed (Fig. 10C). This was not further investigated, as the RecA-UmuC cross-linking appeared most prominent in all experiments and levels of cross-linking to UmuD' were not conducive to downstream mass spectrometry analysis. Cross-linking between RecA and UmuC was also observed prominently in inactive complexes formed in the absence of RecA* (S1 Fig).

**Fig 11:**
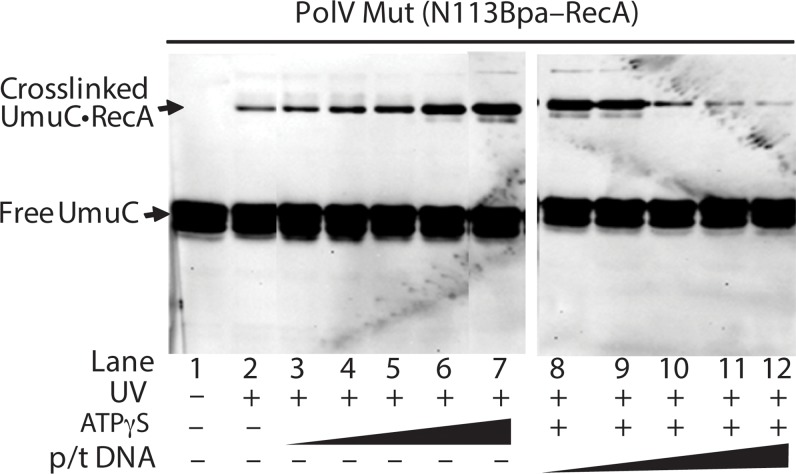
Effects of ATP and primer-template binding on RecA-UmuC cross-linking efficiency in pol V Mut. Western blot using an antibody to UmuC demonstrating increased UmuC-RecA cross-linking with increasing ATPγS concentration and decreasing UmuC-RecA cross-linking with increasing primer-template concentration in the presence of 500 μM ATPγS. pol V Mut was generated using RecA N113Bpa as described in Methods, with ATPγS and primer-template hairpin DNA added where indicated. ATPγS concentration ranges from 0.8 to 500 μM and primer-template DNA concentration ranges from 0.01–5 μM.

In Fig. 10, the level of RecA-UmuC cross-linking appears to increase in the presence of ATP or ATPγS, and decrease in the presence of the primer-template substrate. This is explored further in Fig. 11. Here, the RecA-UmuC cross-linked species clearly increases in prominence as the concentration of ATPγS increases. It then decreases as primer-template DNA is added in a concentration-dependent manner. The results suggest the presence of conformational changes associated with the ATP binding and DNA binding steps in the activation pathway as outlined in Fig. 8. This result also suggests that the crosslinking of RecA N113Bpa to Pol V is not a random or non-specific interaction, as it can be varied as a function of conditional changes that are relevant to the formation of active pol V Mut.

To explore the surfaces on UmuC to which RecA 113Bpa was cross-linked, the UmuC cross-linked peptides were first identified, and then specific amino acid residues within them. RecA-UmuC bands cut out of an SDS-PAGE gel like that in Fig. 10 were subjected to in-gel proteolytic digestion. Digestion only with trypsin produced cross-linked peptides that were too large for convenient analysis, so a double digest with trypsin and AspN proteases was employed. The resulting peptide mixtures were analyzed by high resolution nanoLC-MS/MS.

**Fig 12:**
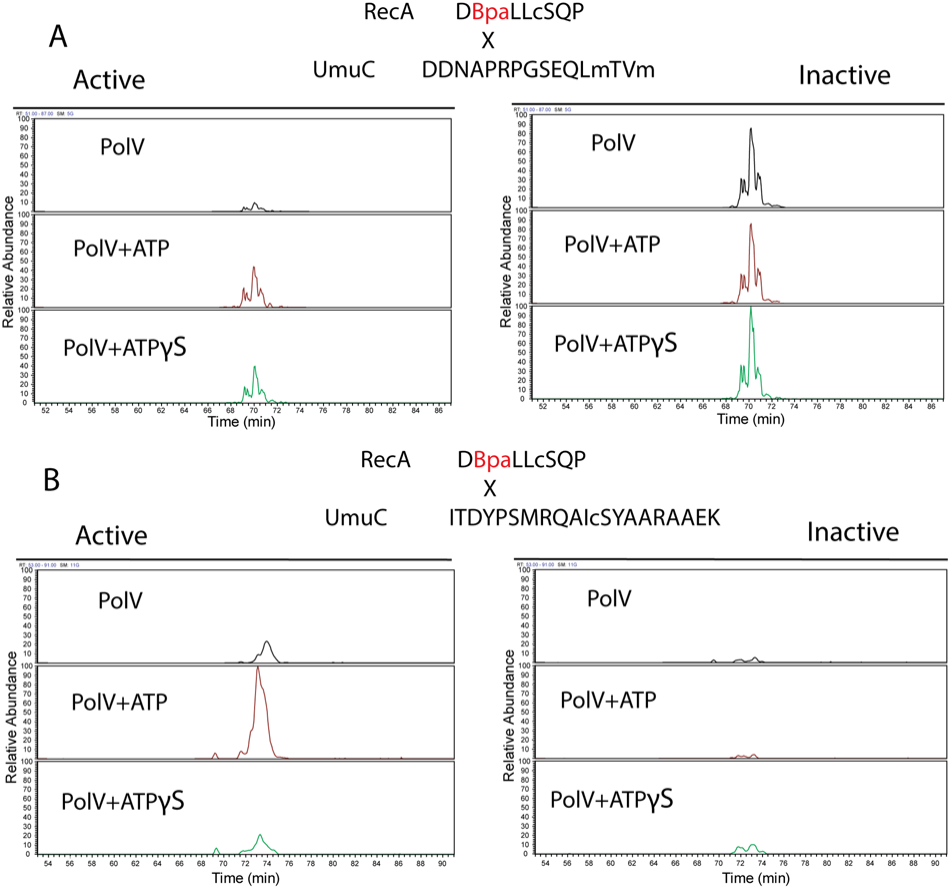
Extracted Ion Chromatograms for the RecA-UmuC cross-linked peptides. (A) Cross-linked product #1. This was the most prominent cross-linked product and was found in virtually all samples generated with either of the two protocols (active or inactive). The product appeared to be somewhat more prominent in the inactive samples. (B) Cross-linked product #2. This product was more prominent in the active samples. Signal intensity was normalized to the highest for each unique peptide pair within a panel (A or B). However, the panel scales are different. Peaks in panel B are approximately 10 fold less prominent than those in panel A.

**Fig 13:**
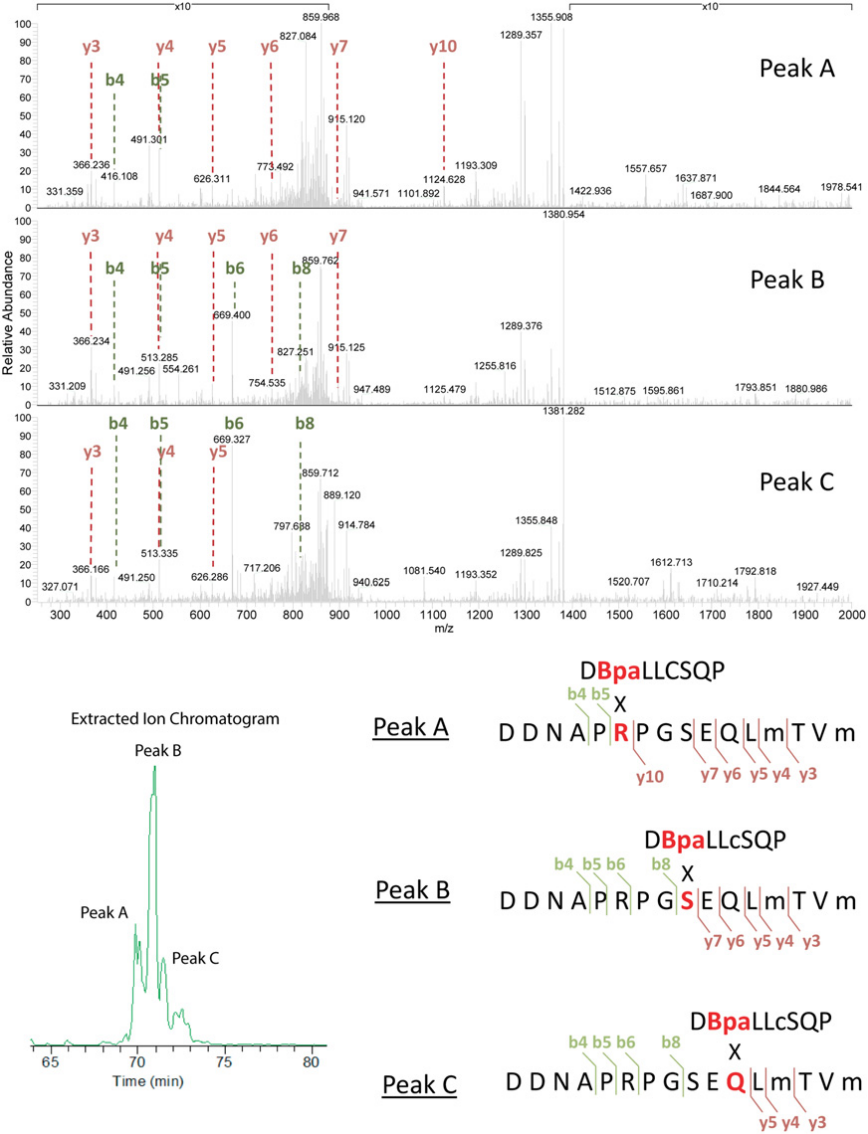
Analysis of cross-linked RecA-UmuC product #1. The first of the two identified cross-linked products appeared in samples generated via both active and inactive pol V protocols, although it seemed to be somewhat more prominent in the inactive samples. The UmuC peptide involved in the cross-linking encompasses residues 361–376. MS/MS spectra for each of the three peaks seen in the extracted ion chromatogram (Fig. 12) for the RecA-UmuC cross-linked product are shown at the top. The predicted crosslinking locations for peaks A,B, and C are shown at the bottom. The lettering corresponds to the unique elution profile for the same precursor ion.

**Fig 14:**
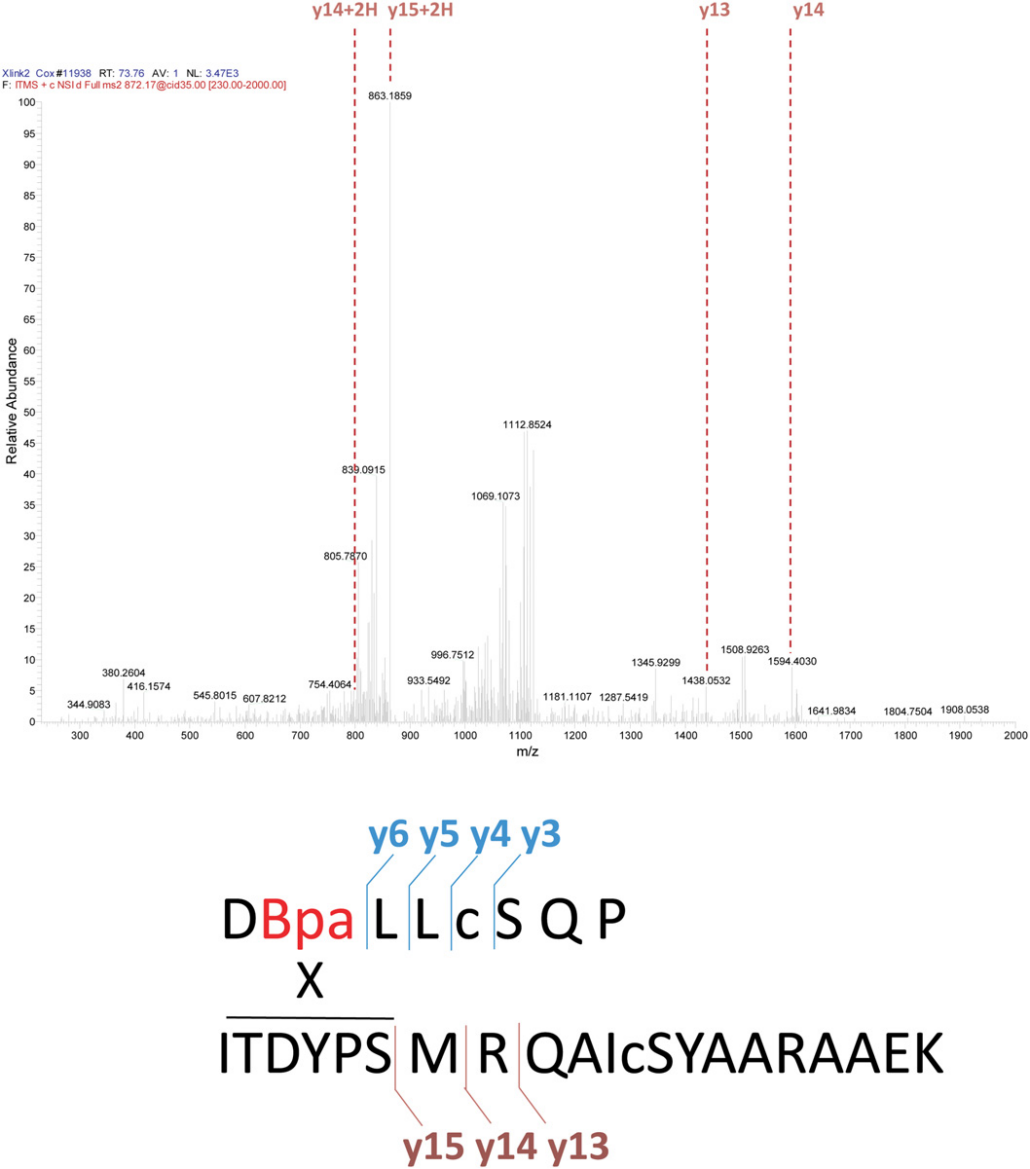
Analysis of cross-linked RecA-UmuC product #2. The second of the two identified cross-linked products appeared primarily in the samples generated via the active pol V Mut protocol, and involved crosslinking between the RecA 113Bpa residue and a peptide spanning residues 256–276 in UmuC. MS/MS spectrum for the RecA-UmuC cross-linked product is shown at the top, with the predicted cross-linking location (the six amino acid residues at the N-terminus, residues 256–261 in UmuC) shown at the bottom.

Two relatively prominent cross-linked products were identified in samples generated by the two protocols. Exemplary MS and MS/MS data for these products are presented in Figs. 12–14. One of these products (Fig. 12A) involved cross-linking to a UmuC peptide extending from residues 361–376. This product appeared in essentially all cross-linked samples, although it appeared to be somewhat more abundant in the inactive samples. This product generally resolved into three ion peaks (Fig. 12A). The second cross-linked peptide was identified unambiguously on the basis of ample representation of this species and its derivatives in the MS and MS/MS data, although it was present in the samples at about 10X less abundance than the other peptide. This involved UmuC amino acid residues 256–276 (Fig. 12B). In this case, the peptide was present most prominently in pol V Mut samples to which ATP had been added. As cross-linking efficiency reflects in part the exact distance between residues to be cross-linked in a given conformation, the abundance of one cross-linked species relative to another is not a direct measure of the abundance of a particular conformation relative to another.

The three peaks for the first product evident in Fig. 12A were subsequently identified as independent cross-link products, reflecting cross-links to UmuC R366, S369, and Q371 (Fig. 13). These cross-link products were present in pol V complexes isolated via both of the two protocols. For the second product, the cross-link was pinpointed to the N-terminal stretch of six amino acids (256–261) within the proteolytically generated UmuC peptide, although the exact amino acid involved could not be unambiguously identified (Fig. 14).

**Fig 15:**
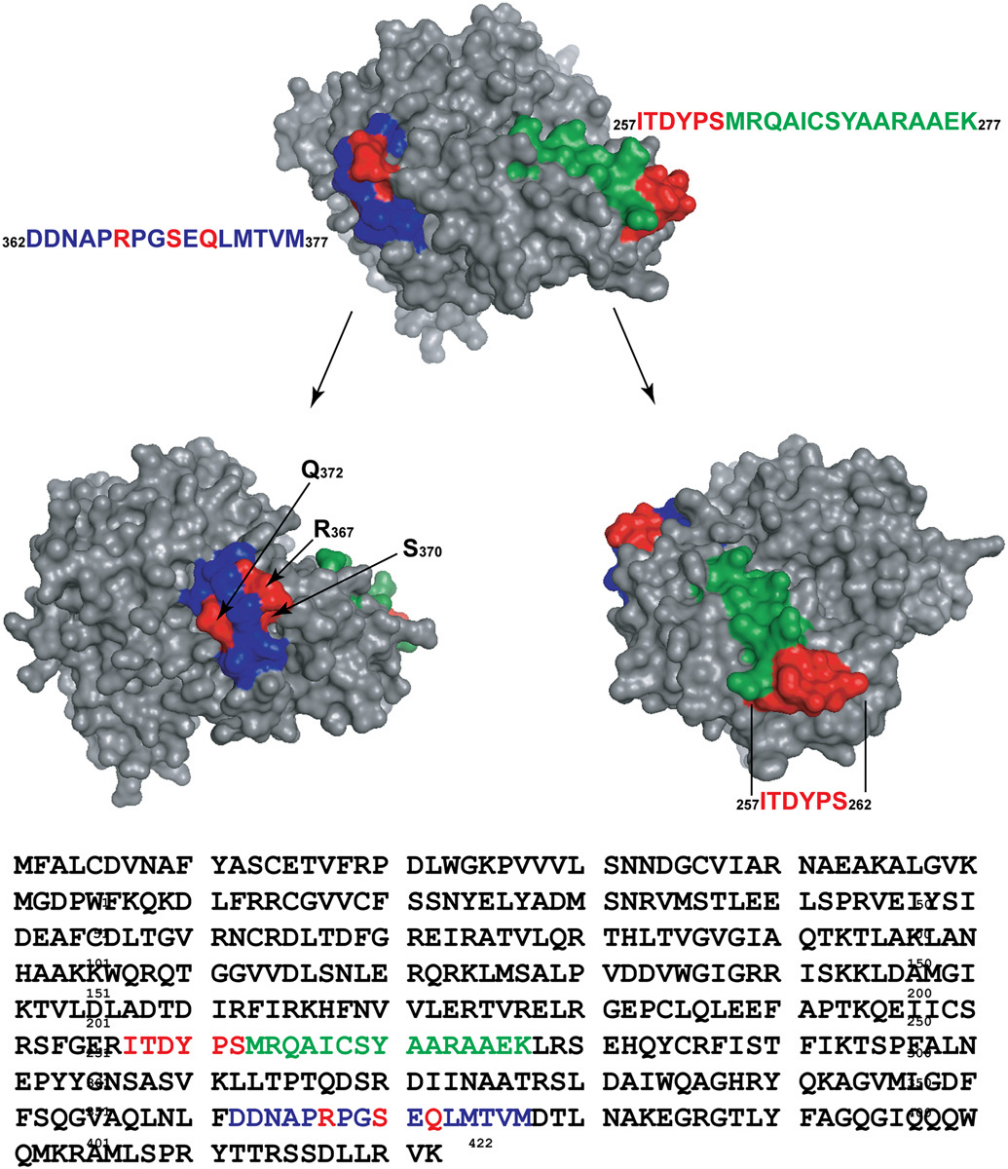
Structural model for UmuC. The model was generated by the program Phyre2 (**P**rotein **H**omology/analog**Y R**ecognition **E**ngine V 2.0) [79] using intensive modeling mode, and exhibits a close structural homology to the known structure of polymerase Dpo4 (S2 Fig). The locations of cross-linked peptides 1 (blue) and 2 (green) are highlighted, with particular amino acid residues involved in cross-linking highlighted in red.

A structural model of UmuC is presented in Fig. 15. This model, generated with the program Phyre [79] overlaps extensively with the known structure of the related polymerase Dpo4 [80](S2 Fig). The peptides identified in Figs. 12–14 are highlighted in color. In the model, both of the peptides include many surface residues. However, the amino acids actually involved in cross-linking within the two peptides (shown in red) are separated by 42.1 to 50.6 Å on the UmuC surface, depending upon which of the amino acid residues identified are used in the calculation. Overall, the results suggest that multiple conformational states are populated in the complexes under study, and again indicate that significant conformational shifts accompany the activation of pol V.

## Discussion

In this work, we begin the task of providing molecular definition to the interaction of RecA* and pol V during pol V activation, focusing first on clues provided by several classic genetic studies [57,58,81]. We conclude that the RecA region encompassing residues 112, 113, and 117 interacts with pol V and represents a RecA surface that is critical to the activation of pol V. The RecA D112R N113R protein is especially deficient in pol V activation, although this variant still forms a complex with pol V. Activation of UmuD′_2_C with RecA D112R N113R is most likely blocked at the step in which ATP binds to the UmuD′_2_C-RecA complex (Fig. 9). Hence, RecA D112R N113R still makes a complex with UmuD′_2_C, but activation does not result. The capacity of this RecA mutant protein to form complexes with UmuD′_2_C implies that the RecA interaction with UmuD′_2_C involves much more extensive surface contacts that must be explored in further studies. This same mutant protein, along with the D112R single mutant, is proficient in essentially all other RecA protein functions.

When a residue of *p*-benzoyl-phenylalanine (Bpa) is substituted for Asn at position 113 in RecA, it is readily cross-linked to UmuC. Bpa is somewhat larger than Asn, but the result implies either a direct interaction or a very close proximity between RecA residue 113 and the UmuC protein. In the conditions tested here, we observed cross-linking at two separate surfaces on UmuC, separated by 42–50 Å from each other. One of these surfaces (involving residues 257–262) is observed reliably only when the pol V is activated via a protocol in which the RecA protein subunit is removed from the end of a RecA* filament, and the RecA* and pol V Mut are separated utilizing centrifugation columns. The result indicates that multiple conformational states are populated in these complexes and that the normal activation pathway may generate some intermediate complexes that are unique to the fully activated state.

Interestingly, the cross-linked UmuC peptide observed in the active pol V Mut is close to UmuC residue 279, previously predicted to interact with the RecA protein [82]. In addition, the surface found most prominently in the inactive state is part of a surface predicted to interact with the replicative β-clamp in the active complex [82]. We speculate that a surface critical to pol V function is therefore occluded by RecA in the inactive complex, while a conformation change in the active pol V Mut serves to expose that same surface for an interaction with the β-clamp.

The RecA D112R and D112R N113R variants form RecA nucleoprotein filaments, hydrolyze ATP, promote DNA strand exchange, and facilitate the autocatalytic cleavage of LexA and UmuD proteins *in vitro*. *In vivo*, these proteins are proficient in UmuD cleavage, induction of the overall SOS response, and recombination function. However, these proteins are specifically deficient in the activation of pol V, both *in vitro* and *in vivo*. In contrast, the classic S117F (*recA1730*) is inactive in strand exchange and exhibits a greatly reduced ATPase activity *in vitro* indicating it is non-functional in some standard RecA activities. It also does not activate pol V. The capacity of RecA S117F to activate RumA′_2_B polymerase clearly indicates that the inability of RecA S117F to activate pol V is not due to any deficiencies in RecA filament formation identified either here or in previous reports. However, we suggest that the RecA D112R N113R protein is an improved substitute for the classic RecA S117F in any study requiring a separation-of-function RecA variant. Except for its defect in the direct activation of pol V, RecA D112R N113R protein is proficient in all other RecA functions assayed.

Several genetic studies have provided evidence that the 3' surface of RecA interacted in some way with pol V, as mutations in residues 112, 113, and 117 suppressed the inhibition of RecA function imposed by overexpression of pol V subunits [57,58,81]. Our work brings these early studies full circle, providing the first molecular evidence that this region is positioned adjacent to or very close to the UmuC subunit of pol V and is essential for the activation of pol V activity. The 3' RecA surface also interacts with the RecA negative regulator, RecX, that caps the 3' RecA so filament extension is obstructed [83]. When RecX was added to reactions with pol V [64], pol V activity was inhibited, further supporting the importance of a 3'-proximal end of a RecA filament in pol V activation.

Our results show that the RecA D112 N113 surface is positioned very closely to UmuC in at least some of its conformational states, and is likely involved in a direct interaction with UmuD′_2_C. The RecA D112R and RecA D112R N113R are still able to interact with pol V, in both the active (Fig. 9) and inactive state (Fig. 6). In the most recent model of pol V activation, RecA was proposed to interact with UmuC in the inactive state and UmuD' in the active state [17,63]. This simple model has been expanded by work presented here, providing greater detail of specific contacts made between RecA and UmuC and molecular changes occurring in the activation cycle of pol V Mut.

We present an updated model for pol V activation (Fig. 8). When pol V first removes a 3'-RecA monomer, RecA 112–117 amino acids are close to UmuC. This is supported by the RecA-UmuC cross-linking in the presence of RecA*. Erdem *et al*. [46] proposed that the RecA/pol V interaction forms the ATPase site, since UmuC and UmuD' do not have an identifiable ATP-binding motif. Possibly the interface between the 3'-RecA surface and pol V, forms the ATPase active site. Only when nucleotide is bound to pol V Mut can it interact with primer-template DNA. It is this ATP binding step in the activation pathway that appears to be blocked in the RecA D112R N113R mutant. Cross-linking declines when the pol V Mut binds to a primer-template (Fig. 11). We propose a shift to RecA-UmuD' interaction (or interaction with both UmuC and UmuD') after binding to a primer-template, based on earlier studies [63] and also on the decline in cross-linking efficiency to UmuC that is observed when the complex binds to primer-template (Fig. 11). DNA pol V Mut is then fully active to synthesize from the 3'-OH. Once synthesis has proceeded far enough, ATP hydrolysis by pol V Mut will dissociate the complex from the primer-template DNA. The released pol V can then be reactivated for another round of synthesis if ssDNA and RecA* are still present.

Pol V activation is not a simple one step mechanism. The RecA N113Bpa has served as an instrumental tool in beginning to map the RecA-pol V interaction upon activation. Future experiments probing the pol V Mut interactions with Bpa incorporated at other RecA positions in the presence and absence of ATPγS, dNTPs, and primer-template DNA, will be necessary to fully understand the conformational shifts and intermediate states in pol V Mut.

## Materials and Methods

### Chemicals and reagents

Potassium phosphate, potassium chloride, magnesium acetate, EDTA, and glycerol were purchased from Fisher. Ammonium sulfate was purchased from MP biomedicals. Dithiothreitol (DTT) was purchased from Research Organics. Tris base was purchased from RPI. Restriction endonucleases and the 2-log DNA molecular weight marker were purchased from New England Biolabs. Chromatography resins were purchased from GE Healthcare except for the ceramic hydroxyapatite, which was purchased from Bio-Rad. A photo-reactive p-benzoyl-phenylalanine, Bpa, was purchased from Bachem. All other chemicals and reagents were purchased from Sigma unless otherwise noted.

### Plasmids and strains

**pEAW242** is *recA* S117F in the overproduction vector pET21d (Novagen). pAIR79 was used as a PCR template with primers consisting of bases 427 to 410 of pET21d and a second primer consisting of bases 370 to 341 of the *recA* gene, with the G at base 353 changed to A to make the complement of *recA* S117F. The PCR product was digested with SgrAI and inserted into pAIR79 SgrAI digest. The resulting plasmid was directly sequenced to confirm the presence of *recA* S117F.

**pEAW739** is *recA* D112R N113R in the overproduction vector pET21a (Novagen). For ease of cloning, the wt *recA* gene was first cloned into pUC19 by digesting pEAW260 at the BglII site before the T7 promoter, and the HindIII site after the end of the *recA* gene, and inserting it into pUC19 digested with BamHI and HindIII. BamHI has compatible cohesive ends with BglII. The resulting plasmid was designated pEAW546. The pEAW260 plasmid was used as PCR template with primers consisting of a NdeI site followed by the first 24 bases of the *recA* gene. The ATG in the NdeI site is also the start codon for *recA*. The other primer consisted of bases 370–327 of the *recA* gene, with the Asp at aa112 and the Asn at aa113 changed to Arg. Bases 263–270 of the *recA* gene code for a SgrAI site. The PCR product was digested with SgrAI and NdeI and inserted into pEAW546 digested with the same enzymes. The resulting plasmid was directly sequenced to confirm the presence of *recA* D112R N113R. The *recA* gene was excised from the plasmid by digestion with NdeI and BamHI, the restriction enzyme site directly following the stop codon of *recA*, and ligated into pET21a digested with the same enzymes. The resulting plasmid was designated pEAW739.

**pEAW861** is *recA* in the overproduction vector pET21a (Novagen) with N113 changed to a TAG stop codon by oligonucleotide mediated mutagenesis for incorporation of the unnatural amino acid p-benzoyl-phenylalanine.

**pHRB1 and pARAR1** are vectors that express His-tagged RumB and untagged RumA' respectively. They were derived from the UmuC expression vector, pHUC25, and the UmuD' expression vector pARAD2 [43] respectively, as follows: pHRB1 was constructed by replacing the PciI—XhoI fragment of pHUC25, which includes *umuC* (excluding His-tag) and *umuD*', with a PCR amplified fragment of *rumB* from pRW320 [52]. pHRB1 therefore expresses His-tagged RumB, i.e. MHHHHHH-MPVFA…LPRVK (1~422 aa of RumB), at a marginal level without a defined promoter. pARAR1 was constructed by replacing *umuD*'of pARAD2 with *rumA*' and it expresses untagged RumA', i.e. M-GFPSP…MRRKS (32~149 aa of RumA), under the control of an arabinose promoter.

**EAW105—WT MG1655 with FRT-Kan^R^**. EAW105 has a mutant pJFS42 FRT-Kan^R^-wt FRT cassette following the *recX* gene on the chromosome of *E*. *coli* MG1655 [84]. EAW105 was constructed using a variation of the procedure of Datsenko and Wanner [85]. A plasmid with the *recX* gene, followed by a pJFS42 FRT-Kanamycin resistance gene-wt FRT and the 212 bp downstream of the end of the *recX* gene was used as a PCR template. The primers consisted of the first 19 bases of the *recX* gene, and bases 212–192 after the end of the *recX* gene. The product was electroporated into MG1655/pKD46, and a Kanamycin resistant colony was selected. PCR was used to confirm the presence of the pJFS42 FRT-Kan^R^-wt FRT after the end of the *recX* gene on the chromosome.

**EAW166—MG1655 *recA* D112R**. EAW166 is *recA* D112R on the chromosome of E. coli MG1655, replacing the wild-type *recA*. EAW166 was constructed in a manner similar to EAW105, with the plasmid used as PCR template consisting of *recA* D112R, *recX* in the wild-type position, the pJFS42 FRT-KanR- wt FRT cassette, and the 218 bp downstream of the *recX* gene. The primers consisted of the first 27 bases of the *recA* gene, and bases 212–192 after the end of the *recX* gene.

**EAW196—MG1655 *recA* D112R N113R**. EAW196 is *recA* D112R N113R on the chromosome of E. coli MG1655. EAW196 was constructed in a manner similar to EAW166, with the plasmid used as PCR template consisting of *recA* D112R N113R instead of *recA* D112R.

**EAW197—MG1655 *recA* D112R N113R L114R**. EAW197 is *recA* D112R N113R L114R on the chromosome of *E*. *coli* MG1655. EAW197 was constructed in a manner similar to EAW166, with the plasmid used as PCR template consisting of *recA* D112R N113R L114R instead of *recA* D112R.

**EAW198—MG1655 *recA* D112R L114R**. EAW198 is *recA* D112R N113R L114R on the chromosome of E. coli MG1655. EAW198 was constructed in a manner similar to EAW166, with the plasmid used as PCR template consisting of *recA* D112R L114R instead of *recA* D112R.

**RW644**-is a BL21(λDE3) derivative with Δ*umuDC596*::*ermGT*, Δ*polB1*::Ωspec and Δ*dinB61*::*ble* alleles [43].

### Proteins

The wild-type *E*. *coli* RecA protein was purified as described [86]. The RecA concentration was determined using the extinction coefficient ε_280_ = 2.23×10^4^ M^-1^ cm^-1^ [87]. The SSB protein was purified as described [88]; its concentration was determined using the extinction coefficient ε_280_ = 2.38×10^4^ M^-1^ cm^-1^. The RecA D112R protein was purified as described [65].

The RecA D112R N113R and S117F proteins were purified using a modified protocol for the RecA D112R protein. All purification steps were carried out at 4°C. Cell paste containing RecA D112R N113R or RecA S117F protein was flash-frozen with liquid N_2_ and then thawed overnight on ice in a solution of 250 mM Tris-HCl (80% cation, pH 7.8) and 25% (w/v) sucrose. The resuspended cells were adjusted to 20% (w/v). The cells were lysed for 45 minutes with a lysozyme solution (2.5 mg/ml final concentration lysozyme in 250 mM Tris-HCl (80% cation, pH 7.8)), followed by addition of 0.4 mL 25 mM EDTA per ml of lysis solution. RecA D112R N113R or RecA S117F protein was precipitated from the lysate supernatant with 0.111 ml of 5% (w/v) polyethyleneimine per ml of lysate. The pellet was washed with R-buffer (20 mM Tris-HCl (80% cation, pH 7.8), 10% glycerol, 0.1 mM EDTA, 1 mM DTT), and RecA D112R N113R or RecA S117F was then extracted from the pellet twice with R-buffer + 150 mM ammonium sulfate. The extracted RecA protein was precipitated with 0.28 g/ml ammonium sulfate and centrifuged. The pellet was resuspended in R-buffer + 1 M ammonium sulfate and then loaded on a 120 mL CV butyl-Sepharose column. A 5 CV linear gradient of R buffer + 1 M ammonium sulfate to R buffer was run and RecA D112R N113R or RecA S117F came off during the gradient. The eluted RecA mutant protein was dialyzed into P-buffer (20 mM potassium phosphate, 10% glycerol, 0.1 mM EDTA, and 1 mM DTT) and applied to a ceramic hydroxyapatite column. RecA D112R N113R or RecA S117F protein was eluted from the column with a 10 CV linear gradient from P-buffer to 1 M P-buffer (same as P-buffer, except with 1 M potassium phosphate). Peak fractions were analyzed by SDS-PAGE. Pooled fractions were dialyzed into R-buffer and applied to a Source 15-Q column. RecA D112R N113R or RecA S117F protein was eluted from the Source 15-Q column with a 10 column volume linear gradient of R-buffer to R-buffer + 1 M KCl. The eluted RecA mutant protein was concentrated by ammonium sulfate precipitation and resuspended in R-buffer + 1 M KCl. The RecA mutant was then applied to a HiPrep 16/60 Sephacryl S-300 HR size exclusion column fractionated by isocratic elution with R-buffer + 1 M KCl. Peak fractions were analyzed by SDS-PAGE. Fractions containing the mutant RecA protein were pooled and dialyzed into R-buffer for storage. The concentration was determined using the wild-type RecA protein extinction coefficient. The purified RecA variants were free of detectable nuclease and polymerase activity.

For RecA N113Bpa, pEAW861 was first transformed into BLR (DE3) (BL21 (*F*−, *dcm*, *ompT*, *hsdS*(*rB*−*mB*−), *gal*, (λ *DE3*), *recA*) cells. This was followed by transformation of pSup-BpaRS-6TRN(D286R), encoding an amber suppressor tyrosyl tRNA and the engineered tyrosyl tRNA synthetase derived from *M*. *jannaschii* for the incorporation of Bpa into the amber codon [89]. Cells were grown at 30°C on LB broth supplemented with 1 mM Bpa to the mid-log phase, and then the expression of amber-mutated RecA N113 were induced by the addition of 0.5 mM IPTG for 4–5 hours. The RecA N113Bpa was purified similar to the above proteins with slightly different chromatography steps. Successive chromatography steps included butyl-Sepharose, ceramic hydroxyapatite, and Source 15Q. The protein was dialyzed into R + 100 mM KCl for storage and concentration was determined using the wild-type extinction coefficient. Molecular weight and incorporation of Bpa were verified by mass spectrometry.

His-tagged RumA′_2_B was purified as previously described for UmuD′_2_C [43] from the *E*. *coli* strain RW644, containing plasmids pARAR1 and pHRB1 (S3 Fig).

### Rifampicin mutation assay

Acquisition of resistance to rifampicin (Rif^s^→Rif^r^ assay) was used to determine mutation rates by fluctuation analysis. Rif^s^→Rif^r^ mutation rates were calculated from 15 tube experiments as follows. A fresh single colony was picked from LB agar supplemented with kanamycin (40 μg/mL), and grown overnight at 37°C with aeration in LB medium. The culture was diluted 1:100 in fresh LB with kanamycin (40 μg/mL) and grown to OD_600_ ~0.3–0.35. Cells were spun down and resuspended in an equal volume of M9 medium. The cells were then treated with 0 J/m^2^ or 60 J/m^2^ using a Spectrolinker XL-1000 UV cross-linker. Cells were spun down again and resuspended in an equal volume of fresh LB and kanamycin and 1 mL aliquots were dispensed into 15 sterile 8 mL culture tubes. Cultures were grown to saturation overnight at 37°C with aeration in the dark. The following day 0.1 mL of each culture was plated on LB agar supplemented with 100 μg/mL rifampicin (Sigma). For wt RecA strains a 1:100 dilution in M9 was made for countable plates. To determine viable cell count, three randomly selected cultures were serially diluted in M9 and plated on LB agar without antibiotics. Plates were incubated at 37°C for 36 hours before counting colonies. The mean numbers of mutations per culture and their confidence limits were obtained with the Ma-Sandri-Sarkar (MSS) maximum-likelihood method [90,91] implemented with the FALCOR web tool found at http://www.mitochondria.org/protocols/FALCOR.html [92], corrected, when required, for plating only 1/10th of the culture. These values were divided by twice the total number of cells per culture to obtain the mutation rates per cell per generation and their confidence limits [93].

### UmuD cleavage *in vivo*

Western blotting of UmuD cleavage to UmuD' was done as previously described [94]. Strains harboring RecA variants on the chromosome where transformed with pJM103 (T7 promoter followed by UmuD, Amp^R^)[95] and pT7 pol26 (Tet^R^) in order to visualize UmuD cleavage. Briefly, a 3 mL overnight with Amp (100 μg/mL) and Tet (50 μg/mL), of each strain was grown at 37°C. A 1:100 dilution of each strain was made the following day in LB with appropriate antibiotics and 0.07 mM IPTG and grown to an OD_600_ 0.3–0.4. Cells were then spun down, resuspended in M9 media, and treated with a UV dosage of 60 J/m^2^ using a Spectrolinker XL-1000 UV cross-linker to induce the SOS response. Cells were spun down again and resuspended in LB, Amp/Tet, and 0.2 mM IPTG. At time points indicated an OD_600_ = 1.0 of each strain was spun down, resuspended in 10 mL of LB, heated for 10 minutes at 95°C, and then treated with DNase I for 15 minutes. An equal cell OD was loaded and run on a 12% SDS-PAGE. The gel was transferred to an Immobilon-P polyvinylidene difluoride membrane (Millipore) at 300 mA for 1 h and blocked in 5% skim milk in PBS-0.1% Tween solution. The membrane was incubated in a 1:1,000 dilution of affinity-purified rabbit anti-UmuD/UmuD' antibodies [95] in blocking buffer. The membrane was washed in PBS and 0.1% Tween and then incubated in PBS-Tween with 1:20,000 diluted goat anti-rabbit horseradish peroxidase secondary antibody (Abcam). The membrane was washed again and visualized with Thermo Scientific SuperSignal West Pico enhanced chemiluminescent substrate on Kodak BioMax light film.

### SOS reporter assay

β-galactosidase assay described in [96] pg 352. An overnight culture of each strain containing the SOS reporter plasmid (pEAW 362 RecN promoter with *lacZ*) was diluted 1:100 into LB with Amp (100 μg/mL). For uninduced SOS, cells were grown to an OD_600_ = 0.28–0.7, chilled on ice, and OD_600_ recorded. Immediately, 0.5 mL cells was added to 0.5 mL Z buffer. To lyse cells, 2 drops of chloroform and 1 drop 0.1% SDS were added. Reaction was started by adding 0.2 mL ONPG in Z buffer at 4 mg/mL and stopped by the addition of 0.5 mL 1 M sodium bicarbonate. An OD_420_ and OD_550_ reading and time to develop color were recorded and used to determine units by the following equation: 1000×(OD_420_–1.75×OD_550_)/time×volume×OD_600_. For induced SOS cells, cells were grown in LB to OD_600_ = 0.4–0.5, spun down, and resuspended in M9 media. The cells were then treated with 30 J/m^2^ using a Spectrolinker XL-1000 UV cross-linker. Cells were spun down again and resuspended in LB + Amp (100 μg/mL) and outgrown for 1 hour. The same protocol as described above was used to measure β-galactosidase activity, but only 100 μL cells were used.

### Strand exchange

The three DNA strand exchange reactions were carried out at 37°C in solutions containing 25 mM Tris-Ac (80% cation, pH 7.6), 10 mM magnesium acetate (Mallincraft), 1 mM dithiothreitol (Research Organics), 3 mM potassium glutamate, 5% (w/v) glycerol and an ATP regeneration system consisting of 2 mM phosphoenolpyruvate (PEP) and 10 units/ml pyruvate kinase. Typically, 3.5 □M RecA and 10 □M css DNA (M13mp18) were pre-incubated in the reaction buffer and regeneration system for 10 min before addition of 3 mM ATP and 1 □M SSB. The reactions were then started by adding 20 □M linear double-stranded (M13mp18) DNA after 10 min of incubation at 37°C with ATP. The reaction was incubated at 37°C for the indicated timepoints. Reaction aliquots (9.5 μl) were deproteinized by addition of a mixture containing 1.2 μl 10% SDS, 0.3 μl 0.5M EDTA and 0.6 μl 20 mg/ml Proteinase K and incubated for 30 min at 37°C. Aliquots were then mixed with 2.5 μl 6 x loading buffer (15% Ficoll, 0.25% bromophenol blue, 0.25% xylene cyanol FF), loaded on a 1% agarose gel, and electrophoresed at 25–35 V for 16 hours at room temperature. To visualize the DNA bands, the gels were stained with ethidium bromide, and exposed to ultraviolet light. Gel images were captured with a digital CCD camera utilizing GelExpert software (Nucleotech).

### UmuD and LexA cleavage *in vitro*

UmuD cleavage was performed essentially as previously described [14]. Standard reaction mixtures (30 μl) contained 40 mM Tris-HCI (pH 8.0), 10mM MgCl_2_, 30 mM NaCl, 2 mM dithiothreitol, 5 μM of M13mp18 circular single-stranded DNA, 3 mM adenosine 5'-(γ-thio)triphosphate (ATPγS), and UmuD, LexA, and RecA as noted. Incubation was at 37°C for the times indicated. The products of the reaction were separated on a 4–15% gradient SDS-PAGE and visualized by staining with Coomassie blue. Where indicated, the intensity of the protein bands was quantitated with the software package TotalLab v1.10 from Phoretix.

### ATPase assay

A coupled, spectrophotometric enzyme assay [8,97] was used to measure the ATPase activities of the wt RecA, D112R, and D112R N113R on circular ssDNA. The regeneration of ATP from ADP and PEP was coupled with the oxidation of NADH and monitored by observing the decrease in absorbance at 380 nm over time. An NADH extinction coefficient of ε_380_ = 1.21 mM^-1^cm^-1^ was used to calculate the rate of ATP hydrolysis. The assays were carried out in a Varian Cary 300 dual beam spectrophotometer equipped with a temperature controller and a 12-position cell changer. The cell path lengths are 1 cm or 0.5 cm and band pass was 2 nm.

The reactions were carried out at 37°C with 25 mM Tris-Ac (80% cation, pH 7.6), 10 mM magnesium acetate, 1 mM DTT, 3 mM potassium glutamate, 5% (w/v) glycerol, an ATP regeneration system (3 mM PEP and 10 U/ml pyrvate kinase), and a coupling system (10 U/ml lactate dehydrogenase, 2 mM NADH for 1-cm cuvettes or 3 mM NADH for 0.5 cm cuvettes). The concentrations of RecA protein, SSB, and DNA, and the time for pre-incubation, are indicated in the Fig legends.

### Fluorescence depolarization assays

The interaction between pol V and RecA variants was probed as described previously [63]. Briefly, wt RecA, D112R, and D112R N113R were labeled using a Fluorescein-EX Protein Labeling Kit (Molecular Probes). Reaction mixtures (110 μL) contained pol V standard reaction buffer (20 mM Tris (pH 7.5), 8 mM magnesium chloride, 5 mM DTT, 0.1 mM EDTA, 25 mM sodium glutamate, and 4% (vol/vol) glycerol), and 20 nM labeled RecA protein. The fluorescence anisotropy was measured at 25°C using a Tecan Infinite M1000 instrument with 470-nm excitation and 535-nm emission wavelengths. All experiments were repeated four times. The average FA values were plotted with one standard deviation of the mean shown as error. Prism software was used to convert FA values to the percent of RecA bound and apparent dissociation (K_d, app_) constants were determined using one-site, specific binding.

### Pol V transactivation assays

Pol V activity was assayed for transactivation by RecA as described previously [64]. Reactions were performed in pol V standard reaction buffer (20 mM Tris (pH 7.5), 8 mM magnesium chloride, 5 mM DTT, 0.1 mM EDTA, 25 mM sodium glutamate, and 4% (vol/vol) glycerol). First the standard reaction buffer with1 mM DTT, 2 mM ATPγS, 2.5 μM poly dT_45_, and 18 μM RecA was incubated at 37°C for 10 minutes to allow RecA filament formation. This was followed by addition of 1mM dNTPs, 1 μM pol V, and 150 nM 65-mer hairpin primer-template DNA which incubated for 60 minutes at 37°C. Reactions (20 μL) were stopped with 40 μL 20 mM EDTA in 95% formamide and ethanol precipitated at-20 °C overnight. Ethanol precipitations were spun down, washed with 70% ethanol and resuspended in 10 μL stop solution. Samples were heated at 95°C for 10 minutes, quick cooled on an ice-water bath for 5 minutes and immediately loaded on a 10% urea denaturing polyacrylamide gel. The synthesis products were imaged using the FAM settings on a Typhoon FLA 9000 (GE Healthcare). Gel band intensities were analyzed to determine primer utilization using ImageQuant TL software (GE Healthcare).

To transactivate of pol V and RumA′_2_B (400 nM each; Fig. 7), 400nM RecA filaments were preformed by preincubating RecA (4 μM) with 30nt ssDNA (0.4 μM) and ATPγS (500 μM) at 37°C for 3 mins in reaction buffer. Subsequently pol V or RumA′_2_B was mixed in with RecA*, 5'- ^32^P-labeled primer-template 3 nucleotide overhang HP (100 nM) and dNTPs (500 μM) to a final volume of 10 μl and incubated for 30 mins (or 1 h for RecA*D112R transactivation) at 37°C. Reactions were quenched in stop solution (90% Formaldehyde and 0.05M EDTA) and resolved on a 20% denaturing polyacrylamide gel allowing single nucleotide separation. Gel band intensities were detected by phosphorimaging and quantified using IMAGEQUANT software.

### Pol V Mut assembly

Pol V Mut was assembled as previously described [46]. Briefly, 5' amino-modified 45nt ssDNA was covalently attached to Cyanogen-Bromide Sepharose resin according to manufacturer’s protocol (Sigma-Aldrich) and transferred into a spin column. Stable RecA* was formed on the ssDNA by mixing RecA (20 μM) and ATPγS (500 μM) then incubating at 37°C for 20 mins. This was followed by the removal of excess RecA and ATPγS via multiple centrifugations of the spin column until no RecA was detected in the flow through. His-tagged pol V (2 nmole) was resuspended in reaction buffer and mixed with RecA*-resin to form pol V Mut. To separate pol V Mut from the RecA*-resin, the spin column was gently centrifuged one last time. The concentration of pol V Mut collected in the flow through was determined by SDS-PAGE gel.

### Binding of pol V Mut to etheno-ATP

Pol V Mut binding to ε-ATP (Life Technologies) was measured as a change in rotational anisotropy. In a 70 μl reaction, ε-ATP was mixed in standard reaction buffer with pol V Mut, pol V + RecA, or RecA (400 nM). Rotational anisotropy was measured using a QuantaMaster (QM-1). Samples were excited with vertically polarized light at 410 nm and both vertical and horizontal emission was monitored at 425 nm.

### Steady-state rotational anisotropy

Binding to p/t DNA was measured by changes in steady-state fluorescence depolarization (rotational anisotropy). Flourescein-labeled 12 nt oh HP (50 nM) was mixed with pol V Mut, pol V + RecA, or RecA alone (400 nM each) and ATPγS was titered to a final concentration of 2000 μM. All reactions (70 μl) were carried out at 37°C in standard reaction buffer. Rotational anisotropy was measured using a QuantaMaster (QM-1) fluorometer (Photon Technology International) with a single emission channel. Samples were excited with vertically polarized light at 495 nm and both vertical and horizontal emission was monitored at 520 nm.

### Formation of active pol V Mut or inactive RecA + UmuD′_2_C complexes for UV cross-linking of RecA N113Bpa to pol V

In order to probe the RecA/pol V interaction, the RecA N113Bpa variant was incubated pol V in several conditions. Pol V-Mut active complex was formed as described above. Once the pol V-Mut was formed, 9.2 μM ATP, 100 μM ATPγS, or 5 μM primer-template DNA and 100 μM ATPγS were added. Different concentrations of ATP and ATPγS were used due to stock concentration limitations of ATP. These mixtures were incubated at 37°C for 5 minutes before samples were subjected to UV light (CalSun unit with four 15-watt bulbs). Samples were kept in 1.5 mL Eppendorf tubes and subjected to 45 mintues of UV at 4 °C. Reactions were separated by SDS-PAGE and stained with Coomassie blue or analyzed via Western blotting for molecular composition of cross-linked complexes.

To form inactive RecA + UmuD′_2_C complexes, reactions contained pol V standard reaction buffer, 1 mM DTT, 5 μM RecA, and 5 μM UmuD′_2_C. When present, ATPγS is at 2 mM and poly dT 45-mer is at 1 μM. It should be noted that pol V storage buffer contains 1 M NaCl, and that the final concentration of NaCl present from addition of pol V is 380 mM. The reactions were incubated at 37°C for 30 minutes before subjected to UV light. Samples were kept in 1.5 mL Eppendorf tubes and subjected to 45 minutes UV at 4°C. Samples were also separated by SDS-PAGE and stained with Coomassie blue or analyzed via Western blotting for molecular composition of cross-linked complexes.

### Enzymatic “In Gel” digestion

“In Gel” digestion and mass spectrometric analysis was done at the Mass Spectrometry Facility (Biotechnology Center, University of Wisconsin-Madison). In short, Coomassie R-250 stained gel pieces were de-stained completely in MeOH/H_2_0/NH_4_HCO_3_ (50%/50%/100 mM), dehydrated for 2 min in ACN/H_2_0/NH_4_HCO_3_ (50%/50%/25mM) then once more for 30 sec in 100% ACN. Dried in a Speed-Vac for 1min, reduced in 25mM DTT (Dithiotreitol in 25 mM NH_4_HCO_3_) for 30 min at 56°C, alkylated with 55mM IAA (Iodoacetamide in 25 mM NH_4_HCO_3_) in darkness at room temperature for 30 min, washed once in H_2_0, dehydrated for 2 min in ACN/H_2_0/NH_4_HCO_3_ (50%:50%:25mM) then once more for 30 sec in 100% ACN. Dried again and rehydrated with 20 μl of trypsin solution with 0.01% ProteaseMAX surfactant (10ng/μl *Trypsin Gold* from Promega Corp. in 25 mM NH_4_HCO_3_/0.01% w/v of ProteaseMAX from Promega Corp.). Let stand for 2 min at room temperature then additional 30 μl of overlay solution (25mM NH_4_HCO_3_/0.01% w/v of ProteaseMAX) was added to keep gel pieces immersed throughout the digestion. The digestion was conducted for 3 hrs at 42°C then peptides generated from tryptic digestion were transferred to a new Protein LoBind tubes (~50 μl volume) and secondary digestion followed by addition of 5 μl AspN proteinase (20ng/μl endoproteinase AspN from Roche Diagnostics in 25 mM NH_4_HCO_3_). That digestion was conducted for 2 hrs at 37°C. Concurrently, the gel pieces post tryptic digestion were dehydrated with 10 μl of ACN and re-hydrated with 20 μl of secondary digestion mix composed of 5 μl AspN proteinase (20ng/μl endoproteinase AspN from Roche Diagnostics in 25mM NH_4_HCO_3_), 13 μl 25 mM NH_4_HCO_3_ and 2 μl of 0.1% ProteaseMAX surfactant. This digestion was allowed to proceed for 2 hrs at 37°C after which the solution was pulled off and combined with the primary ‘in liquid’ AspN digestion.

Proteolysis was terminated by acidification with 2.5% TFA (Trifluoroacetic Acid) to 0.3% final (10 μl added). Degraded ProteaseMAX was removed via centrifugation (max speed, 10 minutes) and the peptides solid phase extracted (*ZipTip C18* pipette tips from Millipore). Peptides were eluted off the C18 column with 1 μl of acetonitrile/H_2_O/TFA (60%:40%:0.1%) diluted to 30 μl total volume with 0.1% Formic acid and 8 μl was loaded on the instrument.

### NanoLC-MS/MS

Peptides were analyzed by nanoLC-MS/MS using the Agilent 1100 nanoflow system (Agilent) connected to a hybrid linear ion trap-orbitrap mass spectrometer (LTQ-Orbitrap XL, Thermo Fisher Scientific) equipped with an EASY-Spray electrospray source. Chromatography of peptides prior to mass spectral analysis was accomplished using capillary emitter column (PepMap C18, 3 μM, 100 Å, 150x0.075 mm, Thermo Fisher Scientific) onto which extracted peptides were automatically loaded. NanoHPLC system delivered solvents A: 0.1% (v/v) formic acid in water, and B: 99.9% (v/v) acetonitrile, 0.1% (v/v) formic acid at 0.60 μL/min to load the peptides and 0.3 μl/min to elute peptides directly into the nano-electrospray over a 35 minutes 0% (v/v) B to 40% (v/v) B followed by 5 minute 40% (v/v) B to 100% (v/v) B gradient. As peptides eluted from the HPLC-column/electrospray source survey MS scans were acquired in the Orbitrap with a resolution of 100,000 and up to 5 most intense peptides per scan were fragmented and detected in the ion trap over the 300 to 2000 m/z; redundancy was limited by dynamic exclusion. Raw MS/MS data were converted to mgf file format using MSConvert (ProteoWizard: Open Source Software for Rapid Proteomics Tools Development). Resulting mgf files were used to search against *Escherichia coli* database containing target constructs plus common lab protein contaminants. Peptide mass tolerance was set at 20 ppm and fragment mass at 0.8 Da.

### Targeted NanoLC-MS/MS

Assignment of amino acid residues involved in the cross-linking was done using new generation hybrid linear ion trap-orbitrap mass spectrometer (LTQ-Orbitrap Elite, Thermo Fisher Scientific) equipped with an EASY-Spray electrospray source. Chromatography of peptides was conducted under the same conditions as above. As peptides eluted from the HPLC-column MS1 scans from 300 to 2000 m/z were acquired in the Orbitrap with a resolution of 120,000 followed by MS2 fragmentation of 10 peptides from the parent mass list (containing masses observed for candidate peptides in the discovery phase using Orbitrap XL system) and 10 additional most intense peptides from each unique MS1 scan; redundancy was limited by dynamic exclusion. Raw MS/MS data was manually interrogated to precisely map the residue involved in the particular cross-linking.

### Cross-linking assignment

To determine the identity of cross-linked products a targeted database search of raw mass spec data was conducted using only the sequence of proteins used in the cross-linking experiment plus common lab protein contaminants. Cross-linked candidates were generated using StavroX software (StavroX Freeware version 3.4.5 from University of Halle-Wittenberg) with BPA chosen as a cross-linker, Lysine, Arginie and Aspartate with 2 missed cleavages as a protease sites and static Cysteine carbamidomethylation plus variable Methionie oxidation as possible modifications. These candidates were manually evaluated for the quality of MS1 and MS/MS data, matched with their theoretical isotopic and fragmentation distribution and finally cross-checked against the non-UV treated peptide mass spec data from proteins used in the cross-linking experiment. In order to define the residue or approximate the location of cross-linking within the peptide a manual analysis of MS/MS data for isomeric cross-linked species (same cross-linked peptide with differential LC elution profile) was conducted.

## Supporting Information

S1 Fig The RecA 3'-surface interacts with UmuC in inactive pol V complexes. The photo-reactive Bpa was incorporated into RecA at position N113 (RecA N113Bpa) and used to probe RecA/pol V interactions in complexes formed in the absence of RecA*. The reactions contain pol V standard reaction buffer, 1 mM DTT, 5 μM RecA, and 5 μM pol V. When present ATPγS was at 2 mM, and poly dT 45-mer was at 1 μM. The reactions were incubated at 37°C for 30 minutes before subjected to UV light. (A) Coomassie stained SDS-PAGE of samples. UmuC, RecA, and UmuD′ bands are indicated. Higher molecular weight bands appear upon crosslinking via UV light. The major crosslinked species runs at ~100 kDa. (B) UmuC western blot of reactions presented in (A). UmuC is present in the major crosslinked band at ~100 kDa. (C) UmuD′ western blot of reactions presented in (A). No specific cross-linking species are visible, indicating RecA N113Bpa does not interact with UmuD′ under the conditions tested.
(TIF)

S2 Fig Structural relationship of the model for UmuC to the polymerase Dpo4. The structural model for UmuC generated by the program Phyre2 is shown above, alongside the known structure of Dpo4 [80] (pdb identifier JX4). The two structures are merged in the lower image. Conserved amino acid residues in the active site of both enzymes are highlighted in purple for UmuC (D101, E102, and steric gate amino acids F10 and Y11) and magenta for Dpo4 (D105, E106 and steric gate amino acids F11, Y12). UmuC peptides cross-linked to RecA 113Bpa are in green (aa 257–277) and blue (aa 362–377), with particular amino acids involved in crosslinking highlighted in red.
(TIF)

S3 Fig A: Restriction map of pHRB1 and pARA1. B: SDS gel of RumA'B purification. SDS-PAGE gel of His-RumB/RumA' purification using Ni-NTA chromatography. Lanes labeled *Un* and *In* are uninduced or induced whole-cell extracts respectively. Lanes *S* and *I*, are the soluble and insoluble fractions respectively. Lanes *FT* and *E*, are the flow-through fractions and fractions eluted from Ni-NTA agarose respectively. Lane *H*, the final preparation after elution from the Hydroxyapatite column.
(TIF)
