# Supplementary figures and images for: A RecA Protein Surface Required for Activation of DNA Polymerase V
Source: PLoS Genet. 2015 Mar 26;11(3):e1005066. doi: 10.1371/journal.pgen.1005066 (PMC4374754; doi:10.1371/journal.pgen.1005066)

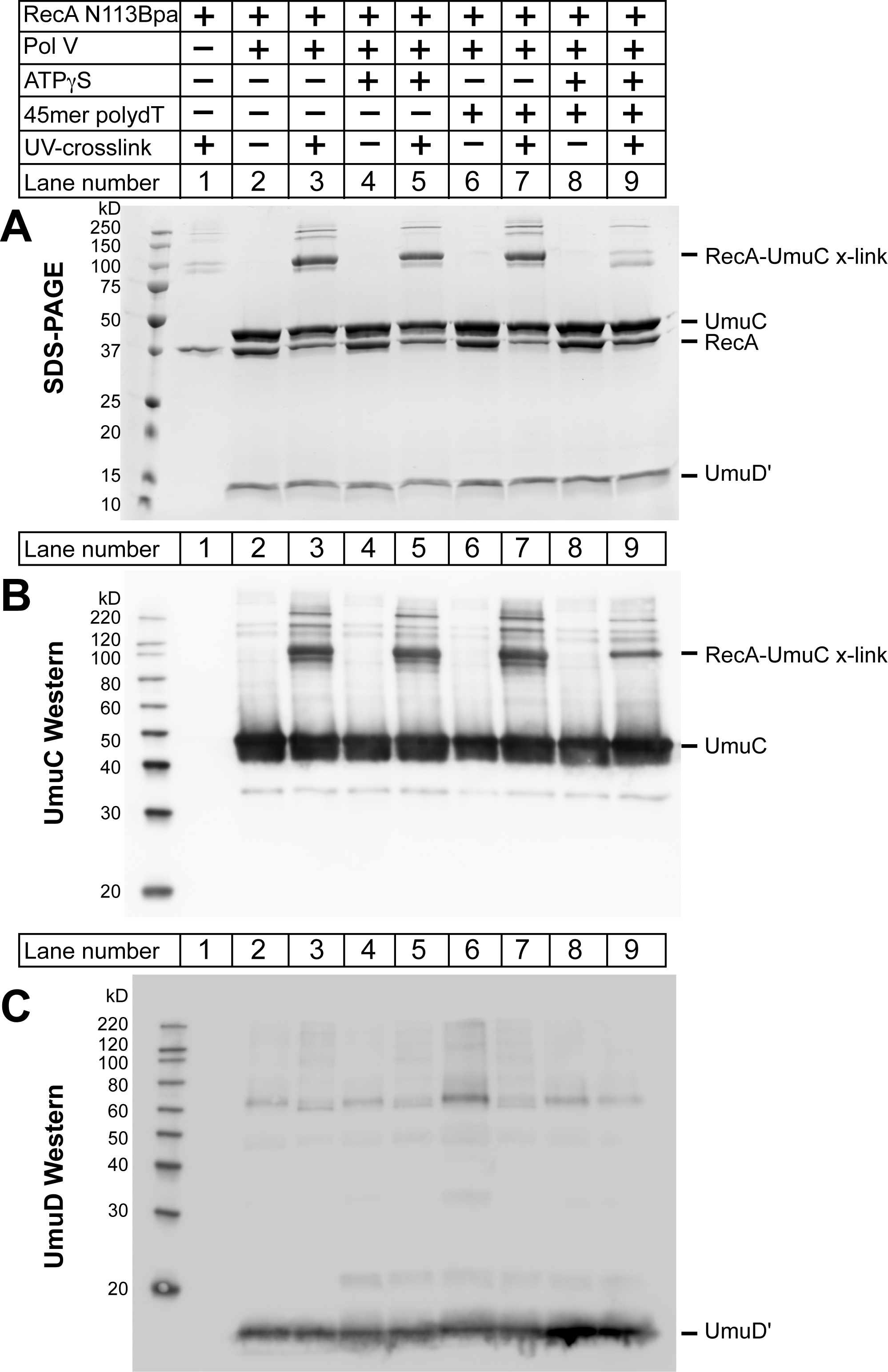

Supplement: S1 Fig — The photo-reactive Bpa was incorporated into RecA at position N113 (RecA N113Bpa) and used to probe RecA/pol V interactions in complexes formed in the absence of RecA*. The reactions contain pol V standard reaction buffer, 1 mM DTT, 5 μM RecA, and 5 μM pol V. When present ATPγS was at 2 mM, and poly dT 45-mer was at 1 μM. The reactions were incubated at 37°C for 30 minutes before subjected to UV light. (A) Coomassie stained SDS-PAGE of samples. UmuC, RecA, and UmuD′ bands are indicated. Higher molecular weight bands appear upon crosslinking via UV light. The major crosslinked species runs at ~100 kDa. (B) UmuC western blot of reactions presented in (A). UmuC is present in the major crosslinked band at ~100 kDa. (C) UmuD′ western blot of reactions presented in (A). No specific cross-linking species are visible, indicating RecA N113Bpa does not interact with UmuD′ under the conditions tested. (TIF) [file pgen.1005066.s001.tif]

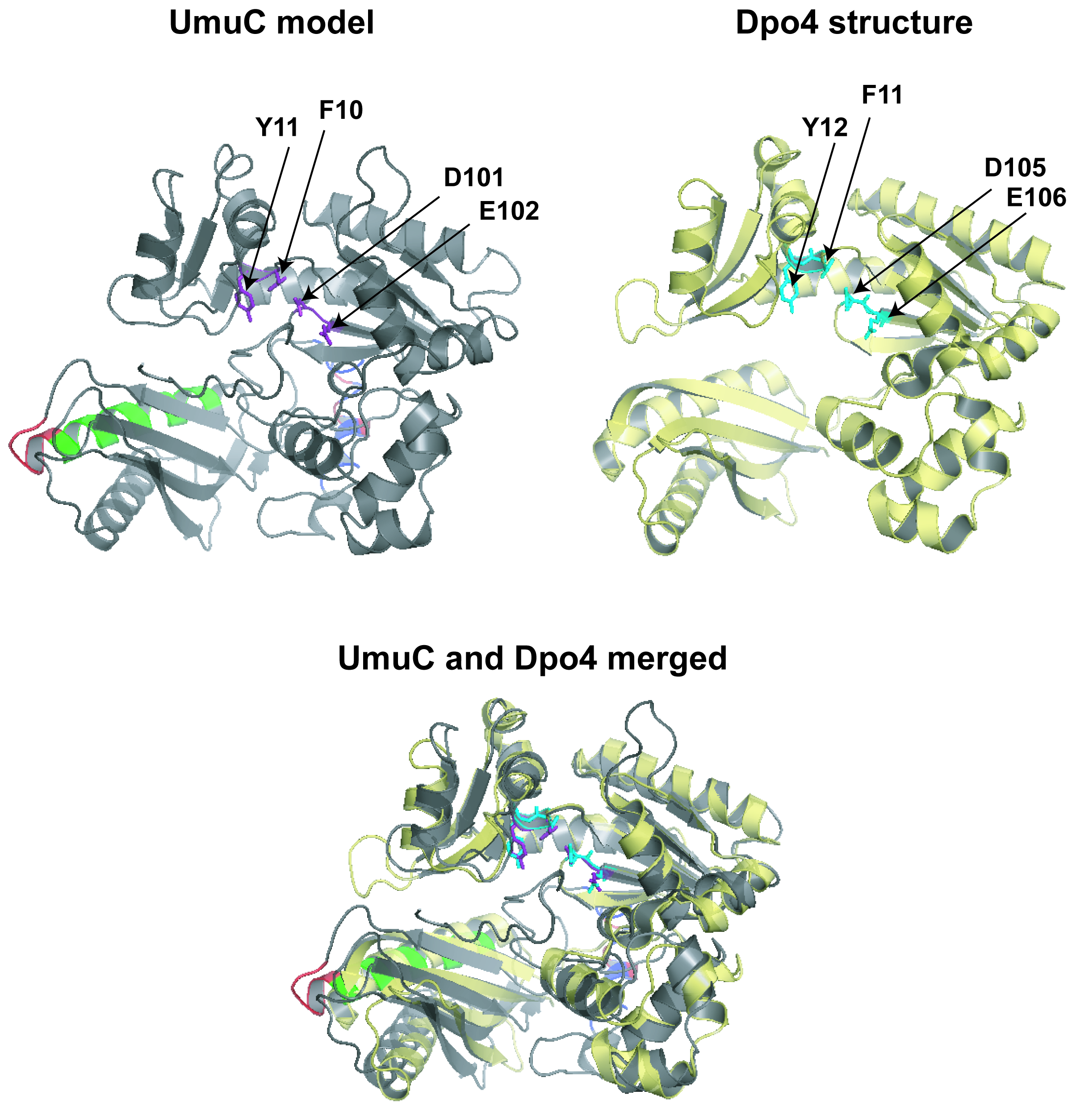

Supplement: S2 Fig — The structural model for UmuC generated by the program Phyre2 is shown above, alongside the known structure of Dpo4 [80] (pdb identifier JX4). The two structures are merged in the lower image. Conserved amino acid residues in the active site of both enzymes are highlighted in purple for UmuC (D101, E102, and steric gate amino acids F10 and Y11) and magenta for Dpo4 (D105, E106 and steric gate amino acids F11, Y12). UmuC peptides cross-linked to RecA 113Bpa are in green (aa 257–277) and blue (aa 362–377), with particular amino acids involved in crosslinking highlighted in red. (TIF) [file pgen.1005066.s002.tif]

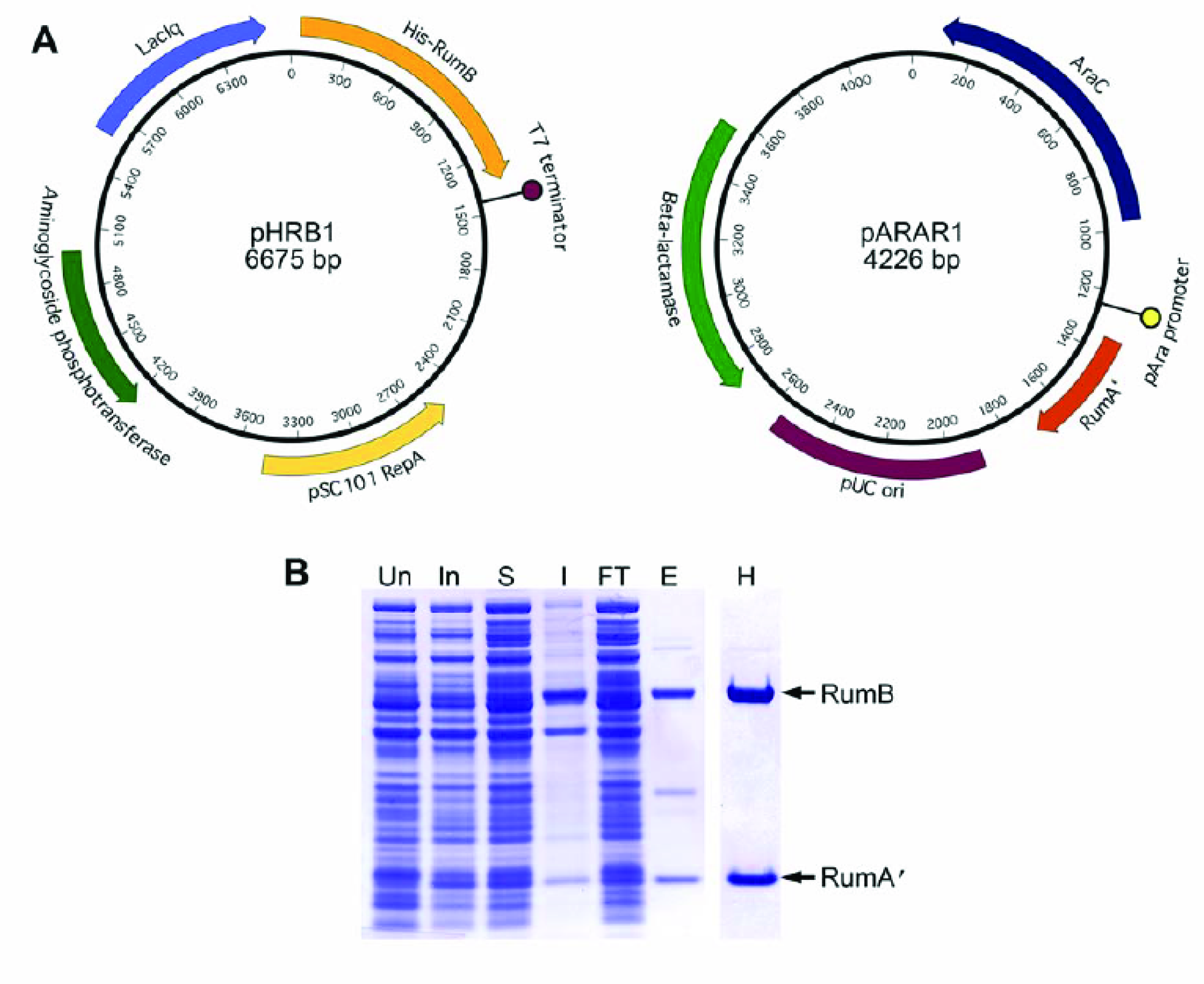

Supplement: S3 Fig — B: SDS gel of RumA'B purification. SDS-PAGE gel of His-RumB/RumA' purification using Ni-NTA chromatography. Lanes labeled Un and In are uninduced or induced whole-cell extracts respectively. Lanes S and I, are the soluble and insoluble fractions respectively. Lanes FT and E, are the flow-through fractions and fractions eluted from Ni-NTA agarose respectively. Lane H, the final preparation after elution from the Hydroxyapatite column. (TIF) [file pgen.1005066.s003.tif]
